# Antibiotic combination efficacy (ACE) networks for a *Pseudomonas aeruginosa* model

**DOI:** 10.1371/journal.pbio.2004356

**Published:** 2018-04-30

**Authors:** Camilo Barbosa, Robert Beardmore, Hinrich Schulenburg, Gunther Jansen

**Affiliations:** 1 Evolutionary Ecology and Genetics, Zoological Institute, Kiel, Germany; 2 Biosciences, Geoffrey Pope Building, University of Exeter, Exeter, United Kingdom; Pennsylvania State University, United States of America

## Abstract

The spread of antibiotic resistance is always a consequence of evolutionary processes. The consideration of evolution is thus key to the development of sustainable therapy. Two main factors were recently proposed to enhance long-term effectiveness of drug combinations: evolved collateral sensitivities between the drugs in a pair and antagonistic drug interactions. We systematically assessed these factors by performing over 1,600 evolution experiments with the opportunistic nosocomial pathogen *Pseudomonas aeruginosa* in single- and multidrug environments. Based on the growth dynamics during these experiments, we reconstructed antibiotic combination efficacy (ACE) networks as a new tool for characterizing the ability of the tested drug combinations to constrain bacterial survival as well as drug resistance evolution across time. Subsequent statistical analysis of the influence of the factors on ACE network characteristics revealed that (i) synergistic drug interactions increased the likelihood of bacterial population extinction—irrespective of whether combinations were compared at the same level of inhibition or not—while (ii) the potential for evolved collateral sensitivities between 2 drugs accounted for a reduction in bacterial adaptation rates. In sum, our systematic experimental analysis allowed us to pinpoint 2 complementary determinants of combination efficacy and to identify specific drug pairs with high ACE scores. Our findings can guide attempts to further improve the sustainability of antibiotic therapy by simultaneously reducing pathogen load and resistance evolution.

## Author summary

Bacterial infections are commonly treated with a combination of antibiotic drugs. However, not all combinations are equally effective, and success is variable. One reason for this variation is that we usually do not know to what extent bacteria are able to adapt to different types of drug combinations. If they can and do adapt, then antibiotic resistance can spread, potentially aggravating the current antibiotic crisis. In the current study, we therefore asked whether combination therapy can be improved by considering the evolutionary potential of the bacteria. To address this question, we systematically assessed the efficacy of antibiotic combinations using controlled laboratory evolution experiments with the opportunistic human pathogen *Pseudomonas aeruginosa* as a model. We found that 2 factors consistently increase treatment efficacy. First, synergism between the combined drugs (i.e., the 2 drugs enhance each other’s effects) increases the rate of bacterial population extinction and thus clearance rate. Second, evolved trade-offs such as collateral sensitivity (i.e., evolution of resistance to one drug increases susceptibility to the other drug) limit the ability of bacteria to adapt to the antibiotic pair. Our findings may help to optimize combination therapy by focusing on drug pairs that interact synergistically and also lead to evolved collateral sensitivities.

## Introduction

The rise of antibiotic resistance is reducing the arsenal of available drugs to treat bacterial infections [1–3]. Some infections are already nearly untreatable because the infecting pathogens are resistant to virtually all available drugs [4,5]. The identification and establishment of new antibiotics has become a major focus of national and international health programs, and substantial investments have been directed towards drug discovery, for example, by the United States and the European Union [6–10]. Yet even if these attempts succeeded and dozens of novel compounds became available tomorrow, the antibiotic crisis would not subside. The evolution of resistance is inevitable, and new drugs will be incapacitated within short time periods [2,3]. So how can we hamper this evolutionary march towards resistance? To some extent, we cannot escape the open-ended arms race between compound discovery and resistance evolution. Nevertheless, we may still use evolutionary thinking to enhance treatment efficacy and sustainability [11]. Combination therapy, the simultaneous deployment of 2 or more drugs, is commonly proposed [12]. Indeed, WHO has endorsed it as the first-line strategy to treat diseases such as tuberculosis, malaria, or HIV [13–15]. However, the nature of the drug combination is crucial for treatment success because initially effective combinations may maximize selection for antibiotic resistance [16,17].

The approach of experimental evolution has proven highly informative on exploring the dynamics that shape the emergence and spread of drug resistance [11,18]. Using this approach, drug pairs were previously suggested to be most effective at limiting bacterial adaptation if (i) antimicrobials display collateral sensitivity, such that bacteria that evolve resistance to one of the compounds immediately suffer exacerbated suppression by the other [19–22], or (ii) antibiotics interact antagonistically, such that they inhibit each other’s effect [16,23,24]. A mathematical model indicated that the latter empirical findings may not be generally applicable but depend on the exact conditions during evolution [25]. In particular, synergistic drug pairs generally favor bacterial clearance but only sometimes low adaptation rates. The strong reduction in population size by synergistic drugs decreases the likelihood of resistance mutations emerging and increases the chances of population extinction. However, these effects only correlate with low adaptation rates when resource competition is weak. When resource competition is high, resistance mutations have a strong selective advantage and may spread rapidly through the population due to competitive release. Under these conditions, antagonistic rather than synergistic drugs are most efficient in reducing adaptation rates [25]. To date, few experimental data are available to explore these particular model predictions—and, moreover, test the role of evolutionary trade-offs, such as the evolved collateral sensitivities—on bacterial adaptation in multidrug environments.

In the current study, we performed a systematic analysis using an experimental evolution approach and the gram-negative opportunistic human pathogen *Pseudomonas aeruginosa* as a model. We evaluated 38 drug pairs for their ability to effectively constrain bacterial adaptation in multidrug environments and calculated 2 antibiotic combination efficacy (ACE) networks based on either the rate of adaptation or bacterial clearance (i.e., frequency of population extinction). These measures provide complementary information on treatment efficacy. First, population extinction represents the ultimate aim of any antibiotic intervention; its frequency is a highly informative indicator of treatment efficacy under our specific experimental conditions, in which antibiotics are always applied at sublethal doses. Second, for the surviving populations, we further evaluated increases in growth rates as a measure of the bacteria’s adaptive potential in antibiotic environments [16]. We subsequently employed complementary statistical approaches, including an integrative Bayesian network (BN) analysis, to disentangle the relative impacts of drug interaction type and evolved collateral effects between individual drugs on the characteristics of the inferred ACE networks. For selected drug pairs, we additionally explored to what extent adaptation to the combinations is driven by the single-component drugs or by initial drug inhibitory levels.

## Results

### Most tested antibiotics interact synergistically in *P*. *aeruginosa*

**Fig 1:**
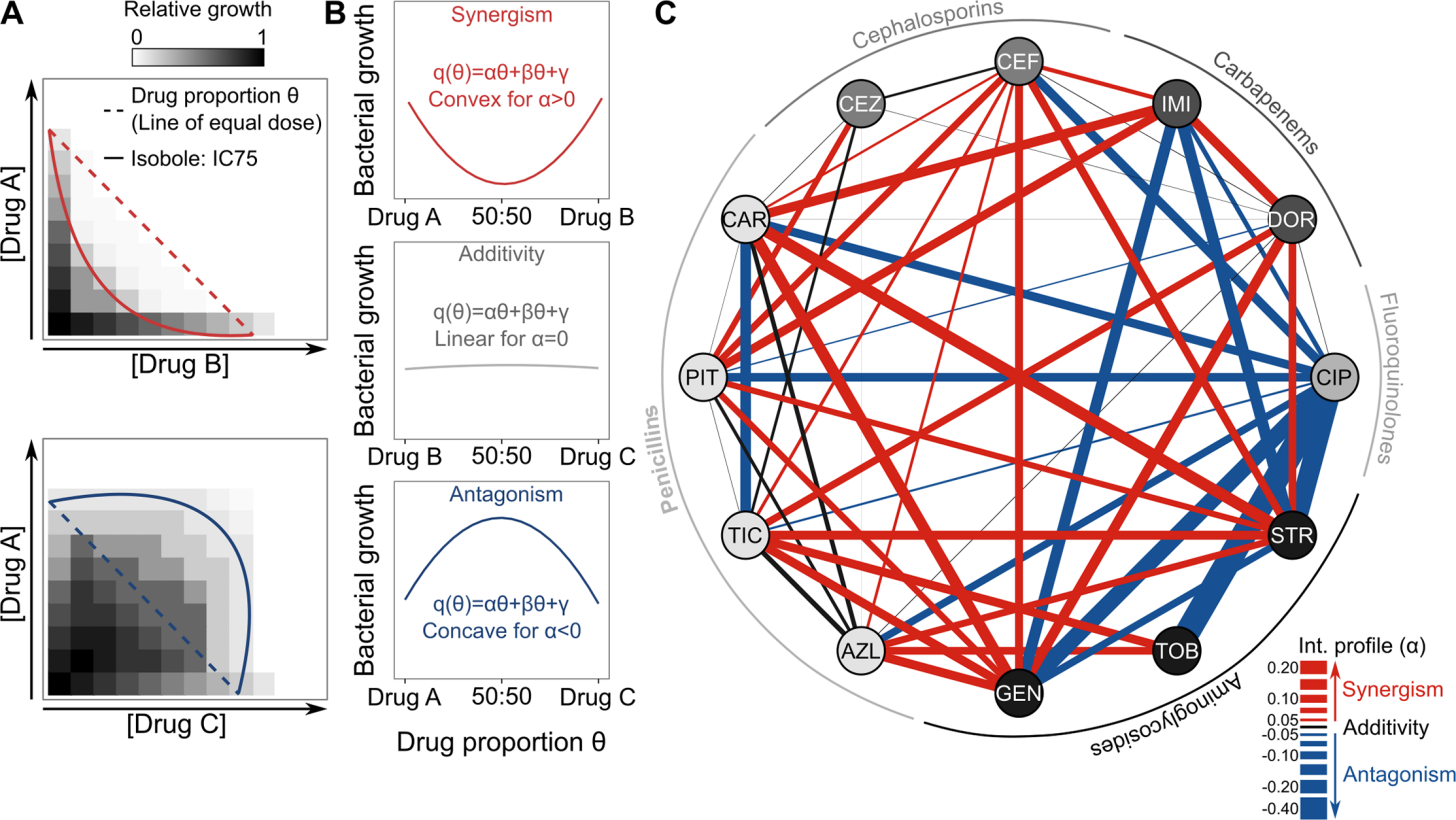
Drug interaction network for *P*. *aeruginosa*. (A) Schematic representation, adapted from [17], of the principle underlying the drug proportion parameter θ (line of equal dose; dashed lines), which is subsequently used to determine drug interactions, in comparison to different shapes of isobolograms (solid lines), as observed in synergistic (in red; top panel) or antagonistic (in blue; bottom panel) interactions. (B) Schematic illustration of the different interaction types as a function of the drug proportion parameter θ, ranging from synergism to antagonism. Drugs are combined in 9 different proportions (*n* = 9 for each combination), with each drug alone set to inhibit 75% of growth (S1 Fig). After a fixed time (12 h), bacterial growth is measured, and a quadratic model is used to fit the observed data. The α test [17] was used to determine significance of synergism or antagonism (S1 Table). (C) The α parameter was inferred from measured data to reconstruct a drug interaction network including 52 different antibiotic combinations. Combinations were formed from 12 different drugs, here represented as the nodes of the network, spanning 5 different antibiotic classes (see outer ring). The drug interaction profile is shown through the links (lines) formed between the nodes, and its strength is highlighted by the thickness of the lines and color. Red, black, and blue lines correspond to synergistic, additive, or antagonistic interactions, respectively (see also S3 Fig). The data for this panel are provided in S3 Data. AZL, azlocillin; CAR, carbenicillin; CEF, cefsulodin; CEZ, ceftazidime; CIP, ciprofloxacin; DOR, doripenem; GEN, gentamicin; IC75, concentration inhibiting 75% of bacterial growth; IMI, imipenem; PIT, piperacillin + tazobactam; STR, streptomycin; TIC, ticarcillin; TOB, tobramycin.

Antibiotic interactions are defined as synergistic, additive, or antagonistic when the drug pair has a stronger, equivalent, or weaker inhibitory effect on bacterial growth than the corresponding single drugs (i.e., monotherapies), respectively. Here, we determined this interaction quantitatively using an estimator denoted α [17]. This estimator is obtained from a quadratic regression applied to growth measurements as a function of different drug proportions of 2 drugs. The concentration of each of the single drugs is chosen to fall onto the line of equal dose, in our case defined to inhibit 75% of growth (i.e., inhibitory concentration [IC] 75; Fig 1A, S1 Fig and Table 1). The estimator α describes the shape of the resulting response in growth whereby positive values indicate synergism and negative values antagonism (Fig 1B). This approach has two advantages: first, it provides a statistical framework for testing the significance of positive or negative α; and second, its inference is less laborious than alternative procedures, thus facilitating characterization of a larger number of drug interactions. Even though the approach was carefully evaluated previously [17], we specifically validated its suitability for our model system. We compared the inferred α values for 8 selected combinations (S2A Fig and S3 Fig) to the corresponding results obtained with one of the commonly used alternative methods, based on Bliss independence and the checkerboard approach (S1 Data for a key to all datasets and S2 Data), as previously described for *Escherichia coli* [16,26]. This comparison demonstrated that α correlates significantly with the degree of synergy (*S*), irrespective of whether *S* is calculated from the average of all viable concentrations across a grid defined by the 2 drugs (AB_*ij*_ = A_*i*_ + B_*j*_; S2B Fig) or from combinations for which the 2 individual drugs had the same level of inhibition (AB_*ij*_ for which IC50_[A*i*]_ = IC50_[B*j*]_, S2C Fig). We thus conclude that the α estimator provides an informative, quantitative indicator of a 2-drug interaction.

**Table 1:** List of antibiotics used in this study.

Functional target	Class	Drug	Abbreviation
DNA repair	Fluoroquinolones	Ciprofloxacin	CIP
Protein synthesis	Aminoglycosides	Tobramycin	TOB
		Gentamicin	GEN
		Streptomycin	STR
Cell wall synthesis	Penicillins	Piperacillin + tazobactam	PIT
		Azlocillin	AZL
		Ticarcillin	TIC
		Carbenicillin	CAR
	Carbapenems	Doripenem	DOR
		Imipenem	IMI
	Cephalosporins	Ceftazidime	CEZ
		Cefsulodin	CEF

We subsequently evaluated the interactions among 12 different antibiotics representing 5 classes (Table 1). We chose these drugs as representatives of the main classes of antibiotics, which are commonly used in combination to treat *P*. *aeruginosa* and to which most clinical *P*. *aeruginosa* strains are still susceptible [27–29]. Even though this choice could have introduced a bias in the overall pattern of inferred interaction types, these should nevertheless be representative of the clinically applied drug combinations. We characterized drug interactions for almost all of the possible combinations, resulting in a total of 52 measures that we summarized in an interaction network (Fig 1C, S3 Fig, S1 Table, S3 Data). Overall, synergistic combinations were more common than other interaction types (synergistic = 24/52; additive = 14/52; and antagonistic = 14/52). Combinations between cell wall inhibitors (β-lactams) and aminoglycosides most often produced synergisms, whereas those including ciprofloxacin (CIP) had exclusively antagonistic effects (Fig 1C).

### ACE networks demonstrate substantial variation in the effect of combinations on adaptation rates and population extinction

**Fig 2:**
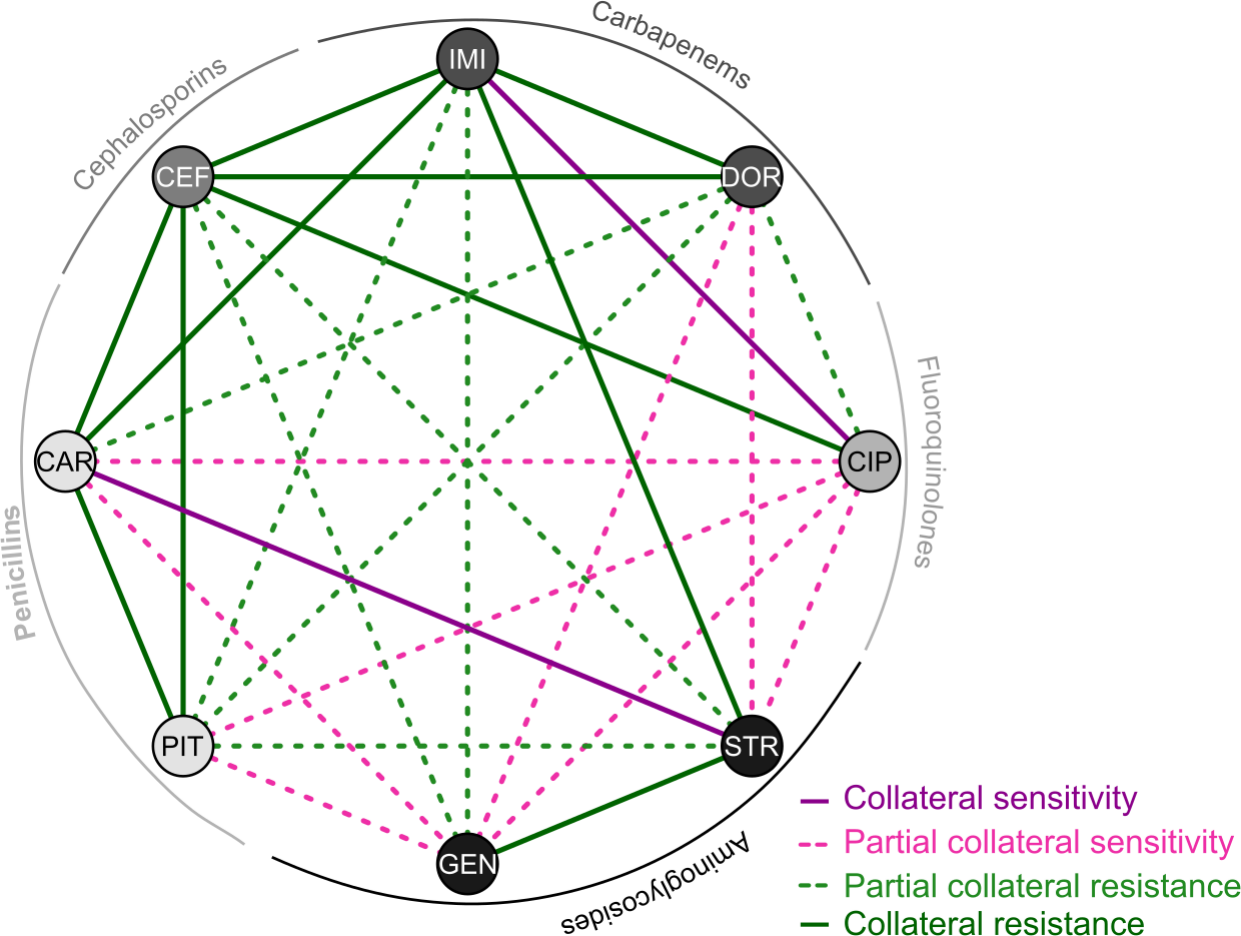
Collateral sensitivity network. The FCRs among 8 of the 12 drugs used in this study were obtained from our previous work [30]. FCR ranges from 0 to 1, such that 0 indicates that all populations (12–20 populations per combination) were sensitive to the corresponding other drug, thus having complete reciprocal sensitivity, whereas 1 highlights that none of the populations with resistance to one of the antibiotics in a pair suffered exacerbated sensitivity against the other. For the graphical illustration, we divided the combinations into 4 groups: complete collateral sensitivity (FCR ≤ 0.25; dark purple lines), partial collateral sensitivity (0.25 < FCR ≤ 0.5; light dashed pink lines), partial cross-resistance (0.5 < FCR < 0.75; light green dashed lines), and complete cross-resistance (FCR ≥ 0.75; dark green lines). CAR, carbenicillin; CEF, cefsulodin; CIP, ciprofloxacin; DOR, doripenem; FCR, frequency of collateral resistances; GEN, gentamicin; IMI, imipenem; PIT, piperacillin + tazobactam; STR, streptomycin.

We used evolution experiments to assess ACE, which is the ability of drug combinations to constrain bacterial adaptation either through population extinction or, in the case of surviving populations, reduced adaptation rates. Based on the inferred drug interactions and the previously obtained frequencies of collateral sensitivity between 8 of the considered antibiotics (Fig 2) [30], we selected 38 drug pairs covering all different types of drug interactions and collateral effects.

**Fig 3:**
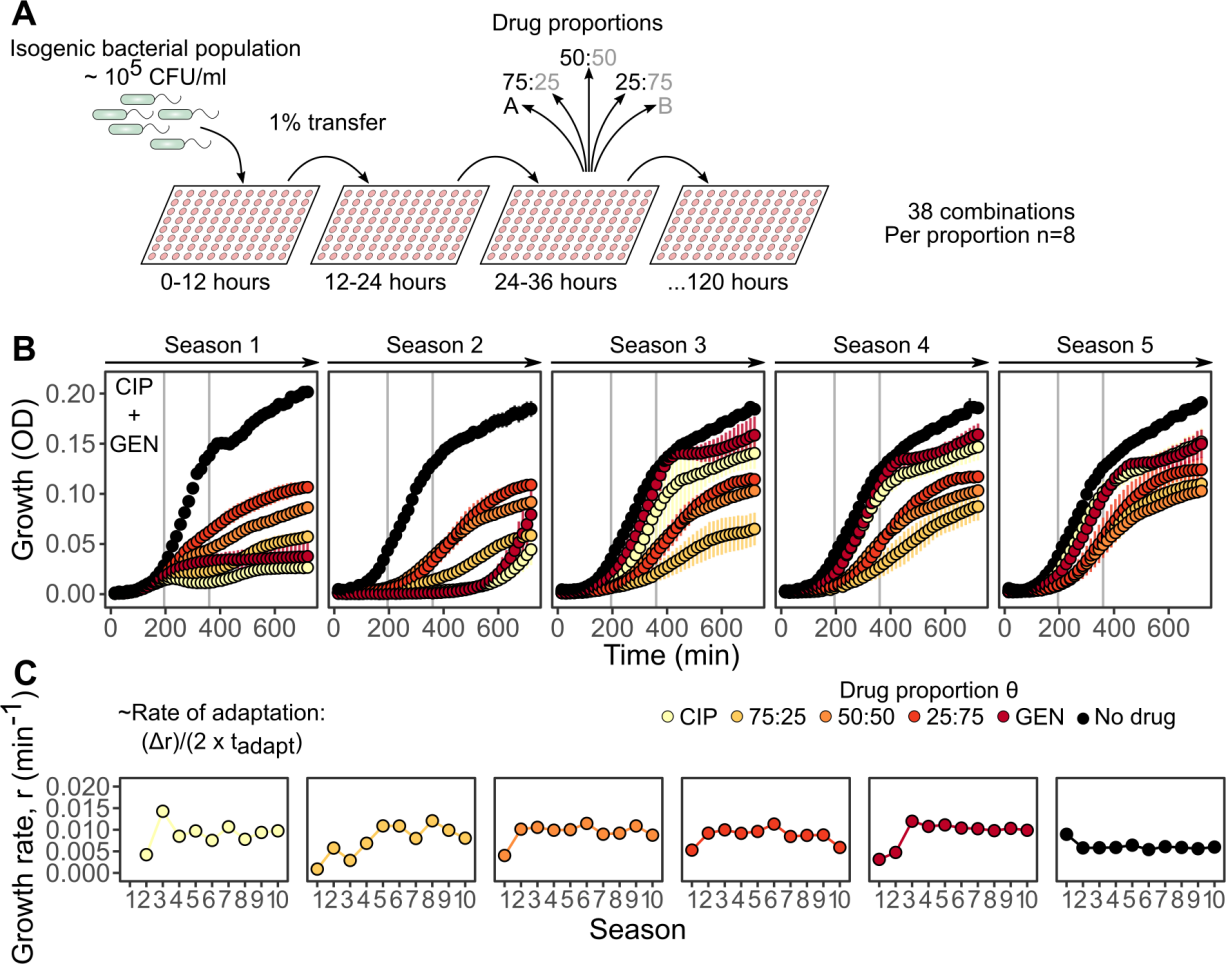
Experimental design and inference of adaptation rates. (A) Schematic representation of the evolution experiment with antibiotic combinations. Thirty-eight combinations were serially transferred every 12 h (season) into fresh medium containing antibiotics mixed in 5 different proportions (*n* = 8 per proportion and drug combination). An uninhibited control was also included, replicated 4 times, resulting in a total of 44 populations per combination and 1,672 for all combinations. Single-drug treatments of any drug A and B aimed at inhibiting 75% of growth relative to a drug-free environment (i.e., IC75). (B) An example of the quantitative growth measures obtained for a particular combination (CIP plus GEN) and the various drug proportions. Each panel shows 1 out of 5 seasons of growth (measured with OD as a proxy ± SD) over a 12-h period. Vertical grey lines denote the time window from which the slope was calculated to infer the growth rate *r* of each evolving population during exponential growth. All the drug proportions considered are highlighted in different colors (yellow to red), as well as the no-drug control (black). (C) Six exemplary populations from the CIP plus GEN combination experiments illustrating the change in growth rate *r* over 10 seasons of growth for each of the drug proportions. The rate of adaptation was calculated following previous work [16], and as indicated on the left of panel C, *t*_adapt_ is defined as the time required to reach half of the change in growth rate, Δ*r*. The data for this figure are provided in S4 Data. CFU, colony-forming unit; CIP, ciprofloxacin; GEN, gentamicin; OD, optical density; IC75, concentration inhibiting 75% of bacterial growth.

Based on this choice of drugs, we evolved a total of 1,672 populations through serial transfers into fresh media containing the respective antibiotics using a transfer period of 12 h and a total of 10 transfers (total duration of 120 h; Fig 3A and S4 Data). We assessed bacterial adaptive potential by integrating quantitative growth measurements taken in 15-min intervals from each evolving population (a total of 783,464 measurements for all treatments and populations; for a validation of our optical density (OD) measures as a proxy for bacterial growth, see Materials and methods and S4 Fig). For each population in a growth season, we then calculated the growth rate *r* during the exponential phase (Fig 3B). Following previous work [16], we defined the rate of adaptation as the change in growth rate over time for each evolving population (Fig 3C; for a validation of using growth characteristics as a proxy of evolutionary adaptation, see Materials and methods and S5 Fig). For subsequent analysis, we focused on the results of the 50:50 drug proportion (S6 Fig) and the single-drug treatments (S7 Fig).

**Fig 4:**
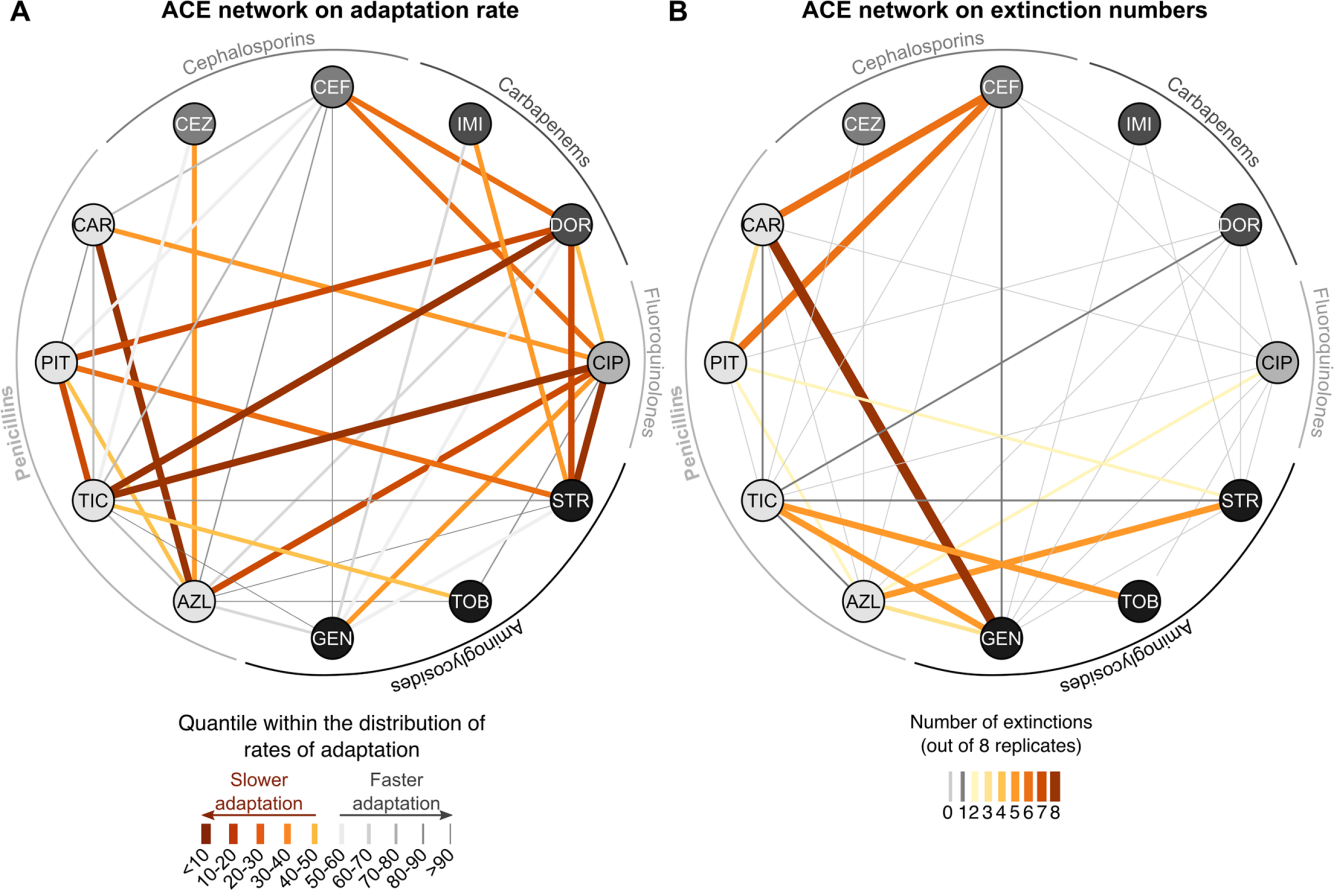
The ACE networks. (A) ACE network built from the rates of adaptation of surviving populations in the combination environment. The color and thickness of the lines (links) formed between the drugs (nodes) reflect the quantiles within which the inferred adaptation rates are found relative to the entire distribution: orange thick lines denote the combinations with the slowest adaptation rates (one of the aims of treatment efficacy), and grey thin lines highlight those with fast adaptation. (B) ACE network on the number of extinction events observed in the combination treatments. Thickness and color of the links represent the number of extinct populations, ranging from 0 (grey) to 8 (dark orange). Adaptation rates and extinction frequencies are inferred from the growth characteristics provided in S4 Data. ACE, antibiotic combination efficacy; AZL, azlocillin; CAR, carbenicillin; CEF, cefsulodin; CEZ, ceftazidime; CIP, ciprofloxacin; DOR, doripenem; GEN, gentamicin; IMI, imipenem; PIT, piperacillin + tazobactam; STR, streptomycin; TIC, ticarcillin; TOB, tobramycin.

We reconstructed the 2 ACE networks based either on adaptation rates of the surviving populations (Fig 4A) or on population extinctions (Fig 4B). Below, we first describe the patterns seen in the ACE networks, while their statistical analysis is explained in the next section. In all cases but one (for carbenicillin [CAR] plus gentamicin [GEN], all populations went extinct), adaptation to the combination treatment was possible. However, the rates of adaptation varied substantially across the different drug combinations, with lower rates of adaptation (below the 50th quantile) predominantly, but not exclusively, seen among antagonistic combinations that included CIP (Fig 4A; S8 Fig and S9 Fig show separate ACE networks for each drug interaction type and the 2 types of evolved collateral effects, respectively). Several synergistic drug pairs, combining an aminoglycoside with either a penicillin or carbapenem, led to similarly low rates of adaptation (below the 50th quantile, S8 Fig). Moreover, almost all cases of collateral sensitivity included in this study were associated with reduced adaptation rates (S9 Fig). This was not the case for combinations with cross-resistance. Furthermore, when estimating clearance efficacy, we found that extinctions almost exclusively occurred with the synergistic combinations (Fig 4B, S8 Fig). The synergistic combinations that did select for lower rates of adaptation did not necessarily have higher rates of extinction and vice versa (populations surviving synergistic combinations were not necessarily adapting more slowly; see azlocillin [AZL] plus streptomycin [STR], cefsulodin [CEF] plus CAR, or ticarcillin [TIC] plus GEN; S8 Fig).

### Statistical ACE network analysis reveals complementary roles for synergism and collateral sensitivity in treatment efficacy

**Fig 5:**
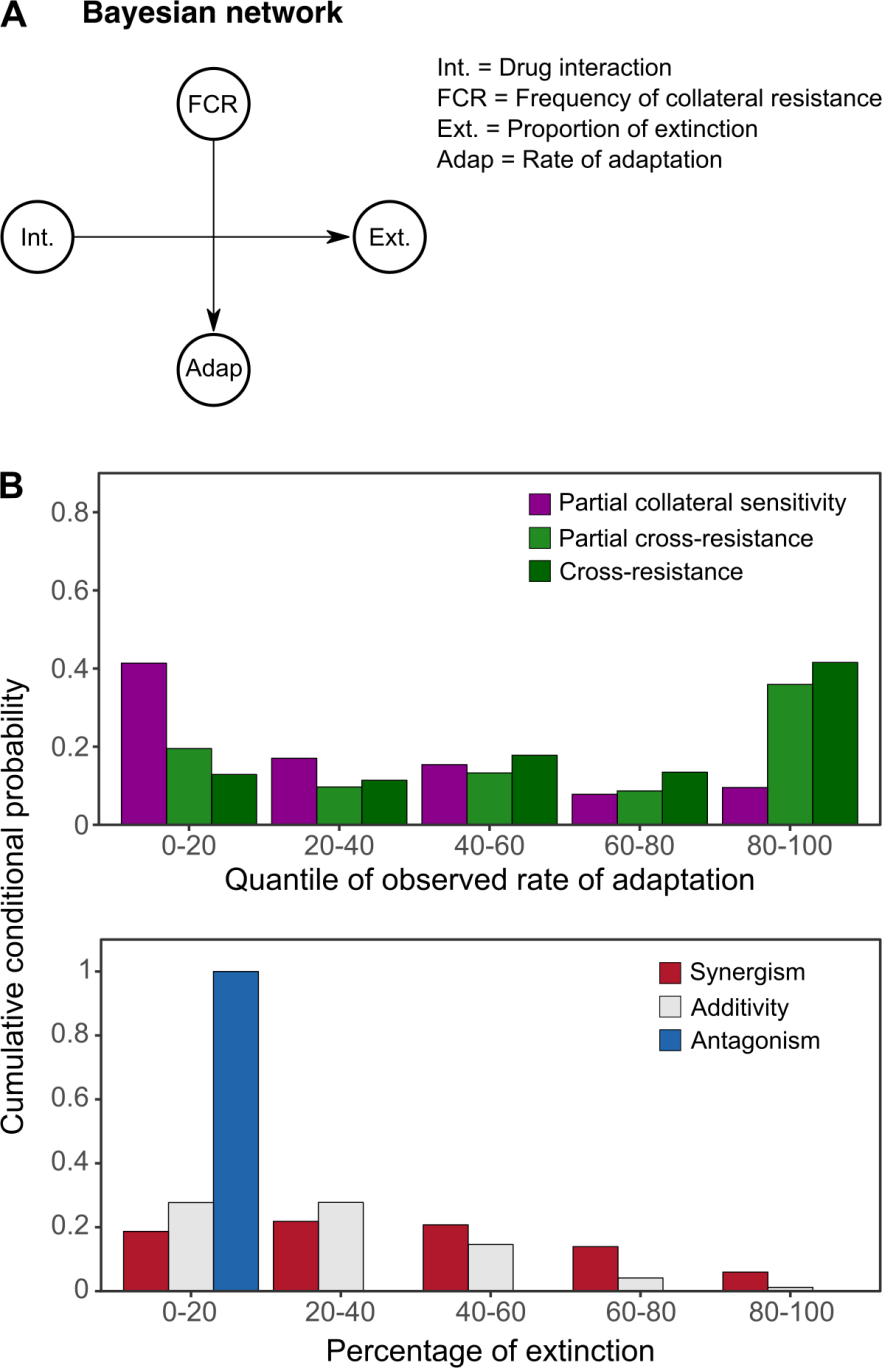
BN analysis of antibiotic resistance evolution under combination therapy. (A) BN obtained from a constraint-based interleaved incremental association algorithm including 4 different random variables: drug interaction types, FCR, proportion of extinctions, and rate of adaptation. (B) Based on the BN, we calculated the conditional probabilities of rate of adaptation for different types of collateral effects (top panel) and extinction frequencies for different antibiotic interaction characteristics (bottom panel). The Bayesian analysis is based on data for drug interaction characteristics (S2 Data), collateral effects [30], and extinction frequencies, and adaptation rates are inferred from growth characteristics during experimental evolution (S4 Data). Adap., rate of adaptation; BN, Bayesian network; Ext., proportion of extinctions; FCR, frequency of collateral resistances; Int., drug interaction types.

We next performed 2 types of statistical analyses to assess to what extent the overall characteristics of the 2 ACE networks are determined by the 2 considered predictors of combination efficacy: interaction type inferred from α (Fig 1C) and collateral sensitivity profiles previously obtained from experimentally evolved resistant populations of *P*. *aeruginosa* (Fig 2, [30]). We first used a BN approach to assess the relationships among the considered variables (i.e., adaptation rate, extinction frequency, drug interaction, and frequency of collateral resistances [FCR]). The BN approach is based on a constraint-based interleaved incremental association algorithm [31–33] to dissect the relationships between our variables (see Materials and methods for details). The results are summarized in the BN (Fig 5A), in which nodes represent the different variables and arrows indicate the inferred dependencies. The BN analysis revealed that the type of antibiotic interaction strongly influenced the proportion of extinction, but not the rate of adaptation. Instead, the rate of adaptation was found to depend solely on the frequency of collateral sensitivities. No other dependency was inferred by the analysis.

Based on the BN structure, we calculated the conditional probabilities for the inferred dependencies between the frequencies of collateral sensitivity and the rates of adaptation as well as for the proportion of extinction and drug interaction type. In particular, we used the different types of evolved collateral effects (i.e., partial collateral sensitivity, partial cross-resistance, and cross-resistance; none of the combinations evaluated during evolution had complete collateral sensitivity between their components, as shown in Fig 2) and calculated the conditional probability of obtaining the distribution of observed adaptation rates across 5 equal quantile bins (Fig 5B, top panel). Similarly, given the different drug interaction types (synergism, additivity, and antagonism), we calculated the conditional probabilities of different extinction frequencies across 5 equal quantile bins (Fig 5B, bottom panel). These 2 additional analyses describe more clearly the inferred dependencies within the BN. Antibiotic combinations for which at least half of the populations had collateral sensitivity against one or both of the individual drug components (i.e., partial collateral sensitivity; purple bars in Fig 5B, top panel) have a higher probability of selecting for low but not high rates of adaptation. Conversely, combinations with partial or complete cross-resistance (green bars in Fig 5B, top panel) have a higher probability of producing the top scores of inferred adaptation rates. In addition, high probabilities of extinction are associated with synergistic and additive combinations, whereas the reverse is found for antagonistic drug pairs (Fig 5B, bottom panel).

We further validated the inferred dependencies between variables using partial correlation analysis, following the approach previously established for a similar analysis of combination efficacy in *E*. *coli* [16]. This approach allowed us to control for drug pair membership using the average rate of adaptation towards the corresponding single drugs of a particular combination as a covariate (Materials and methods). Statistical significance was subsequently inferred using a permutation test [16]. This analysis revealed a significant correlation between the FCR and the rate of adaptation (ρ_s_ = 0.52, *P =* 0.038) and between the proportion of extinction and the drug interaction type α (ρ_s_ = 0.51, *P =* 0.043), but not between the FCR and the proportion of extinction (ρ_s_ = 0.39, *P =* 0.146) or the drug interaction α and the rate of adaptation (ρ_s_ = 0.3, *P =* 0.262). This analysis, based on a distinct statistical approach, thereby corroborated the findings of the BN analysis. We conclude that synergistic drug interactions enhance bacterial clearance, whereas collateral sensitivity limits the adaptive potential of the bacteria.

### Adaptation to the strongest component influences adaptation to multidrug environments

**Fig 6:**
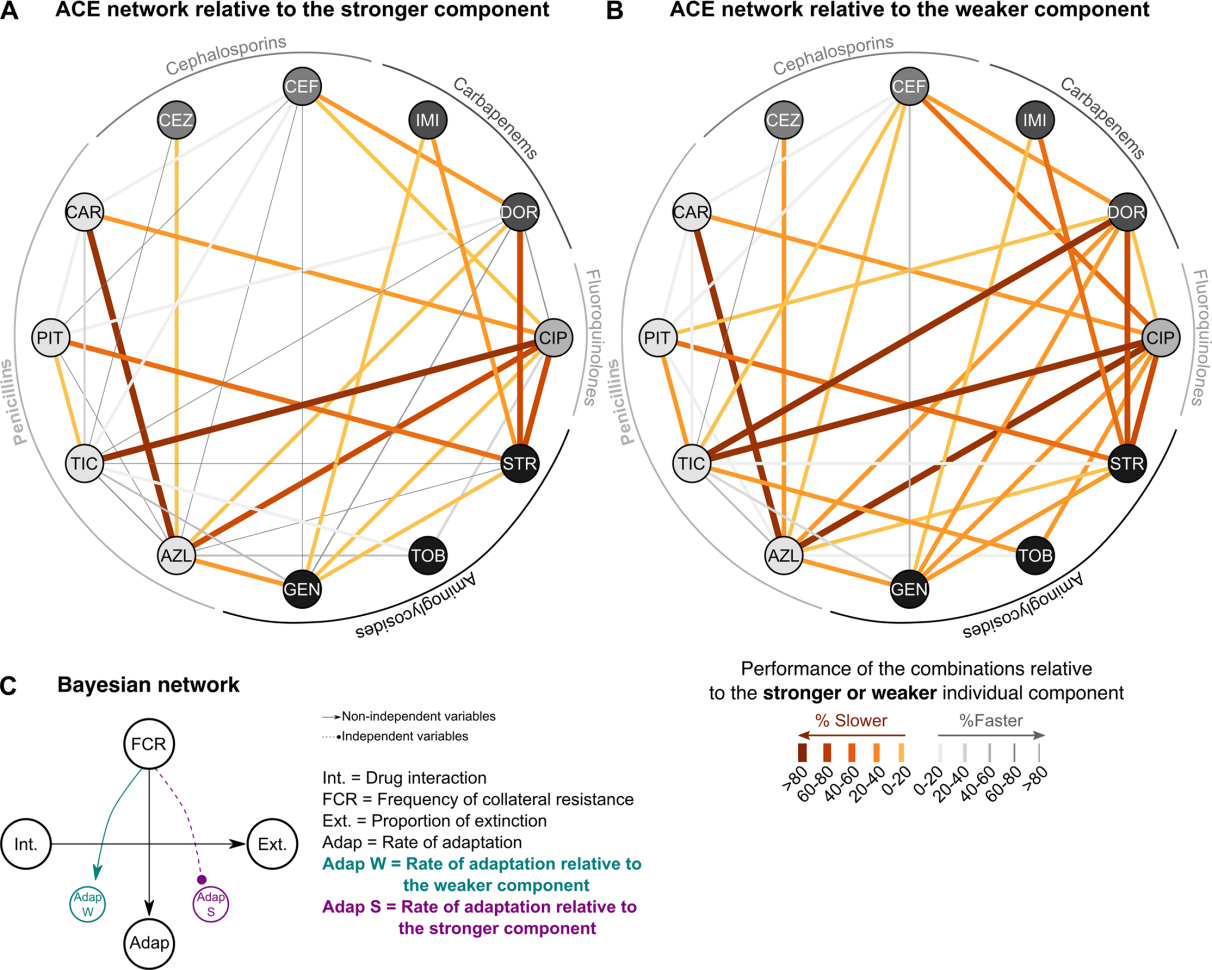
Weighted ACE networks and their Bayesian analysis. We assessed to what extent adaptation to one of the drugs of a pair determined the overall rate of adaptation to the combination treatment. The stronger component drug of each pair was identified as the one with lower adaptation rates in monotherapy. We subsequently standardized the adaptation rates towards the combination by those towards either (A) the stronger or (B) the weaker component drug, resulting in 2 weighted ACE networks. Orange thick lines indicate slower adaptation, while grey thin bands denote fast adaptation. (C) Results of the BN analysis on the original network versus the 2 standardized networks. The relationship between drug interaction type and extinction frequency was stable across all analyses, while the dependence of adaptation rate on evolved collateral effects disappeared when adaptation rates were standardized by the stronger component. Adaptation rates are inferred from the data on growth characteristics during experimental evolution, provided in S4 Data. ACE, antibiotic combination efficacy; AZL, azlocillin; BN, Bayesian network; CAR, carbenicillin; CEF, cefsulodin; CEZ, ceftazidime; CIP, ciprofloxacin; DOR, doripenem; GEN, gentamicin; IMI, imipenem; PIT, piperacillin + tazobactam; STR, streptomycin; TIC, ticarcillin; TOB, tobramycin.

We next assessed whether the ability of bacteria to adapt to the combination is mainly driven by adaptation to only one of the drugs rather than dependent on a unique property of the antibiotic pair. For our dataset, we related the inferred rates of adaptation in the combination treatments to those inferred for the corresponding single-drug environments (S10 Fig). We first compared the 2 corresponding monotherapies of a given drug pair and defined the drug leading to lower rates of adaptation as the stronger component (i.e., higher ability to minimize resistance evolution) and the other as the weaker component (i.e., lower ability to minimize resistance evolution). Thereafter, we calculated the relative rate of adaptation of the combination by standardizing it against either the stronger or the weaker component of the pair. The resulting ACE networks are shown in Fig 6A and 6B, respectively. Interestingly, the original ACE network for adaptation rates (Fig 4A) is more similar to that standardized by the weaker but not the stronger component drug (Fig 6; S2 Table). This suggests that the characteristics of the original ACE network (Fig 4A), and thus the efficacy of drug combinations to reduce adaptation rates, is primarily driven by adaptation to the stronger component, which—if accounted for by the standardizing scheme—removes important properties of the network (see as prominent examples the disappearance of the strong reduction in adaptation rate for doripenem [DOR] plus TIC, or DOR plus PIT [piperacillin + tazobactam]; Fig 4A and Fig 6A).

We further evaluated influence of the component drugs by repetition of the BN analysis. We found that the dependency observed between the FCR and the rates of adaptation of the combinations disappeared when the latter is weighted by the stronger but not the weaker component drug (Fig 6C). At the same time, the dependency between drug interaction and extinction frequency remained, while no additional relationship was revealed. Similar results were obtained when we repeated the correlation analysis with standardized adaptation rates. The originally identified correlation between the FCR and the rate of adaptation was no longer significant when the latter was standardized by adaptation to the stronger component drug (ρ_s_ = 0.33, *P =* 0.21), yet it still showed a statistical trend when we standardized by the weaker component drug (ρ_s_ = 0.45, *P =* 0.078). In these 2 analyses, drug interaction did not correlate significantly with the weighted adaptation rates (ρ_s_ < 0.47, *P* > 0.09). These results consistently indicate that adaptation to the stronger component drug influences adaptation to the combination and that this is dependent on the evolved collateral effects.

### Initial inhibition levels correlate with adaptation rates, while extinction events are almost exclusively restricted to synergistic combinations

We next performed a separate evolution experiment with 4 selected combinations to assess to what extent the inherently different starting levels of inhibition—imposed by each type of interaction during the first season of growth (Fig 1B and S3 Fig)—influenced both the number of extinctions and adaptation rates. We performed this evolution experiment with 4 selected combinations with different interaction profiles: 2 interacting synergistically (GEN plus CAR and STR plus PIT) and 2 antagonistically (GEN plus CIP and Tobramycin [TOB] plus CIP). For these combinations, we varied the initial inhibition level of the combination across 8 steps, ranging from IC50 to >IC90. Populations were serially transferred into fresh media as explained before (S5 Data; and for the obtained changes in growth rate *r*, see S11 Fig).

**Fig 7:**
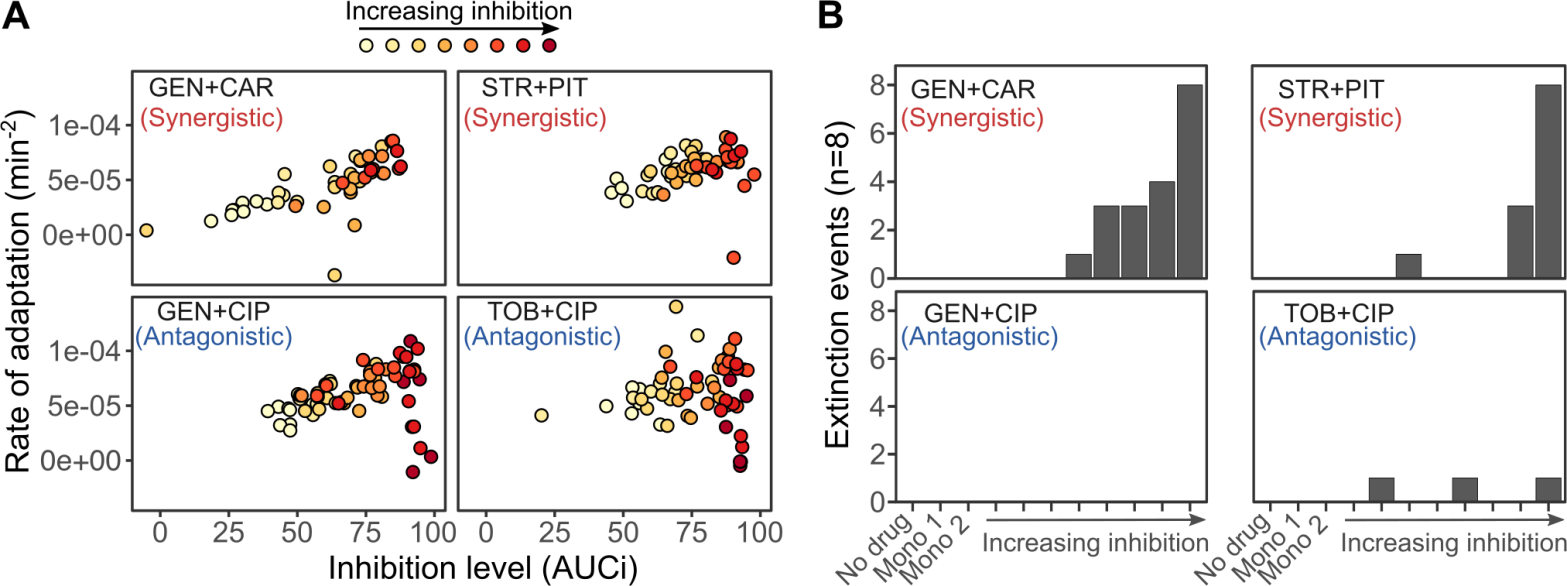
Influence of the initial inhibition level on adaptation rate and population extinction. In a separate round of evolution experiments, we evaluated the consequences of the initial inhibition levels in synergistic and antagonistic combinations. The experiment was performed following the protocol of the main evolution experiment (Fig 3A), with the exception that the starting doses of the combinations were fixed at different levels of inhibition for the combinations. (A) Inferred rates of adaptation as a function of initial inhibitory levels. We determined initial inhibition by measuring in the first season of the evolution experiment the AUC of growth over time (measured as OD as a proxy) for each replicate population and then standardized it against the average AUC of the no-drug control (x-axis, AUCi). The rate of adaptation (y-axis) was inferred as for the main evolution experiment (Fig 3). (B) Extinction was significantly more often observed in synergistic rather than antagonistic combinations, even at the same level of inhibition (results for logistic regression analysis in S4 Table). Adaptation rates and extinction frequencies are inferred from growth characteristics during experimental evolution, provided in S5 Data. AUC, area under the curve; AUCi, area under the curve of relative inhibition of growth; CAR, carbenicillin; CIP, ciprofloxacin; GEN, gentamicin; OD, optical density; PIT, piperacillin + tazobactam; STR, streptomycin; TOB, tobramycin.

This separate evolution experiment revealed that initial inhibitory levels of the tested combinations are significantly related to the rates of adaptation, irrespective of combination identity or drug interaction type (GLM, F_1,336_ = 37.735, *P* < 0.001; Fig 7A and S3 Table). In particular, increasing levels of inhibition are generally associated with higher rates of adaptation, suggesting that strong inhibition increases selection for an adaptive response [34,35]. At higher levels of inhibition, the synergistic and antagonistic combinations produce clearly distinct responses, especially regarding population extinction. Here, the 2 synergistic pairs are associated with a significant increase in the number of extinct populations (logistic regression, *F*_12,336_ = 21.15, *P* < 0.001; Fig 7B and S4 Table), while antagonistic combinations produced almost no extinction at all. Moreover, at the very high initial inhibitory levels, antagonistic pairs showed a sudden drop in adaptation rates (Fig 7A), as expected from previous work [16,24]. A similarly strong reduction is not observed for the synergistic combinations, possibly owing to the fact that only few populations survived and could be used to infer adaptation rates.

Taken together, the results from this separate evolution experiment suggest that the generally higher inhibition levels of the synergistic pairs in our main evolution experiment could potentially have contributed to higher adaptation rates for this type of combination (even though these were not found to be significantly increased compared to those for other interaction types; see above). This seems less likely the case for extinction events, which are generally more frequent in treatments with synergistic rather than antagonistic combinations, irrespective of the initial inhibition level.

## Discussion

Our study provides a systematic experimental analysis of the efficacy of antibiotic combination therapy in the opportunistic human pathogen *P*. *aeruginosa*. Based on evolution experiments with 38 distinct combinations, ACE networks were reconstructed for 2 complementary measures of treatment efficacy: the frequency of population extinctions and the reduction in adaptation rates. Subsequent statistical analyses identified the likely ACE determinants: Synergistic drug interactions enhanced the frequency of extinction, even at the same inhibitory level as antagonistic interactions, while reduced adaptation rates depended on the evolved collateral sensitivities among the drugs. The latter effect is likely driven by adaptation to the stronger component drug in a pair. Consequently, our findings suggest that treatment efficacy against *P*. *aeruginosa* can be optimized by drug combinations, which interact synergistically to increase bacterial clearance and which can evolve collateral sensitivity to each other to slow down the rate of adaptation.

The use of BN analysis enhanced dissection of the determinants of ACE. The BN approach has been widely applied across different fields of biology in recent years but not yet in studies on antibiotic resistance evolution [33,36–39]. Its accessible graphical output and the underlying probabilistic theory facilitate the inference of causal relationships between different variables [31,32]. It further offers estimation of conditional probabilities that reflect the strength of the inferred dependencies; a strategy well suited for the stochastic nature of biological systems and their measurements [40]. The latter is important for the analysis of antibiotic resistance evolution, for which we are mainly interested in anticipating bacterial adaptation based on distinct drug properties or deployment strategies [11,12,41–43]. The suitability of the BN approach for analysis of drug resistance evolution was corroborated with a previously established statistical approach, based on partial correlation analysis [16], which identified a significant relationship for the same pairs of variables.

Our analyses consistently revealed that synergistic drug interactions are an important ACE determinant, especially in terms of bacterial clearance (Fig 4A). The particular importance of bacterial elimination as a component of treatment efficacy was previously considered in a mathematical model [25] but has not yet been evaluated empirically. The previous model assessed the effect of antibiotic interactions on treatment efficacy [25] by modifying a previous infection model based on data from mice infected with *P*. *aeruginosa* [44]. The model is related to the design of our main evolution experiment in that the concentration of a particular drug in a combination is standardized by its inhibitory effect in monotherapy. The model predicted contrasting treatment outcomes for synergistic combinations: On the one hand, synergism enhances extinction, most likely because it strongly reduces population size, thereby decreasing the likelihood of new resistance mutations arising. On the other hand, if resistance emerges, synergism increases the selective advantage of the resistant mutants through competitive release, enhancing bacterial adaptation [25]. Our experimental results are consistent with both alternatives. Although synergism mainly favored bacterial extinction (Figs 5–7), it was in several cases associated with low adaptation rates (Fig 4A). However, in our study, the effect of drug interaction on adaptation rate was always insignificant, irrespective of the analytical approach.

Interestingly, we found higher population extinction for synergistic rather than antagonistic combinations also at low initial inhibitory concentrations (Fig 7). This finding cannot have resulted from the stronger reduction in population size (i.e., inhibitory levels were the same for the 2 interaction types) but must have depended on other properties of the synergistic drug pairs. A likely explanation may be found in the mechanism underlying synergism, which can rely on increased membrane permeability induced by one of the drugs, subsequently enhancing cellular uptake of the second drug [45]. Such mechanisms may have a cumulative effect across time [45] and/or may generally be difficult to counter. This, in turn, limits the number of suitable resistance mutations and ultimately increases the likelihood of extinction. A detailed exploration of this effect clearly warrants further research.

Our experiments further identified the potential to evolve collateral sensitivity as a key determinant of low adaptation rates. This result is generally consistent with previous work on *E*. *coli* and *Staphylococcus aureus* [46,47], although this is the first time it has been shown for *P*. *aeruginosa*. Adaptation rates are thus significantly influenced by evolutionary trade-offs, whereby adaptation to one of the drugs of a pair constrains adaptation to the other. Our findings and those of colleagues [46,47] thereby highlight that such trade-offs may not only improve treatment when drugs are applied sequentially, as originally proposed for evolved collateral sensitivities in *E*. *coli* (i.e., collateral sensitivity cycling; [20–22]). Instead, they can also optimize combination therapy. Our analysis further revealed that the involved dynamics are likely driven by adaptation to the stronger component drug of a pair (Fig 6). This suggests that, if adaptation to the stronger component comes with a higher likelihood of collateral sensitivity to the second drug, adaptation to the combination is systematically slowed down, as, for example, for CIP plus STR or CIP plus CAR (Fig 2, Fig 4A). In contrast, when adaptation to the stronger drug is more likely to cause cross-resistance, then this can enhance adaptation to the combination, as seen for GEN plus STR or CAR plus CEF (Fig 2, Fig 4A). The further exploration of these trade-offs represents a promising avenue to improve treatment efficacy.

Our finding of the high clearance efficacy of synergistic combinations shows some consistency with clinical practice. For *P*. *aeruginosa*, we predominantly observed drug synergism between β-lactams and aminoglycosides (Fig 1C). These 2 antibiotic classes are also most commonly used in combination therapy against this pathogen [29,48,49]. Our results empirically confirm the potency of the β-lactam–aminoglycoside combinations, especially penicillin–aminoglycoside pairs, in causing higher numbers of extinct replicate populations (Fig 4B and S8 Fig). In some cases, the populations surviving these specific combinations also adapted more slowly (e.g., STR plus PIT or TIC plus TOB in Fig 4A and 4B, and S8 Fig). Furthermore, the effectiveness of these combinations may not only be caused by drug synergism but additionally by reciprocal collateral sensitivity that can evolve among these pairs [30]. Our systematic analysis performed under controlled laboratory conditions thus provides empirical support for the often experience-driven choice in clinical treatment. In the future, the clinical applicability of our results should be further explored. For example, we identified high clearance efficacy of certain combinations of penicillins and cephalosporins (Fig 4B) or low adaptation rates if fluoroquinolones (e.g., CIP) were combined with aminoglycosides or penicillins (Fig 4A). It would be of particular interest to corroborate these patterns for clinical isolates in laboratory experiments or under clinical conditions.

In summary, our systematic analysis of antibiotic combinations identified the role of drug interactions and evolved collateral effects in determining 2 complementary properties of treatment efficacy. The comprehensive dataset collected in our study may serve as a useful reference for further exploration of effective therapy, including more detailed statistical analyses such as those that use the potency of pairwise interactions to estimate higher-order drug effects [50,51]. Our approach and the specific results obtained may, moreover, help to improve the design of medical treatment with the 2-fold aim of minimizing pathogen burden and reducing resistance evolution. A similar combined assessment of the efficacy of drug interaction and evolved collateral effects may not only be applicable to other pathogens and infectious diseases. It could similarly help to improve cancer therapy, as previously evaluated for selected cancer types and drug interactions [52–55].

## Materials and methods

### Bacteria and media

All experiments were conducted with *P*. *aeruginosa* PA14. Cells were grown at 37 **°**C in sterile M9 minimal medium supplemented with 0.2% glucose and 0.1% casamino acids. All antibiotics were prepared according to the manufacturer’s instructions and filter sterilized before each experiment (Table 1). All experiments were carried out in randomized 96-well plates shaken and incubated at 37 **°**C in BioTek Eon plate readers, which were also used for regular measurement of ODs in 15-min intervals. Randomization schemes of plates for each experiment were different from each other. All analyses were performed using the R platform (version 3.3.2) unless specified otherwise [56].

### Dose-response curves and minimal inhibitory concentration

We tested 14 different concentrations of each drug in order to establish dose-response relationships after 12 h of incubation. For all concentrations, a 1- to 2-ml 10× stock was prepared and then diluted in a randomized 96-well plate with 6 replicates per concentration, resulting in 90 replicates per antibiotic and 1,080 for all treatments. Ten microliters of an isogenic bacterial population of PA14 were added to a final volume of 100 μl, equivalent to 10^4^ to 10^5^ CFU/ml initial population size. In addition, 2 types of controls were included: one without antibiotic and a second one without both antibiotic and bacteria, each also replicated 6 times. We used a logistic regression to analyze the dose-response relationship of each drug using the package “drc” in R [57]. The obtained models (S1 Fig) allowed accurate calculation of different levels of inhibitory concentrations for each drug, including the minimum inhibitory concentration (MIC; here defined as the concentration inhibiting >90% of growth).

### Checkerboards and degree of synergy

To measure the type of interaction using the checkerboard approach, we considered 9 concentrations of each antibiotic in a pair, including a no-drug control, and distributed them randomly across a 96-well plate. Each pair was evaluated twice. Plates were incubated at 37 °C for 12 h with constant shaking and regular OD measurements taken every 15 min. We then calculated the growth rate *r* for each individual well and combination by fitting a linear regression of growth over time during the exponential phase. Exponential phase was generally observed during 195 to 360 min of each season.

We subsequently determined the degree of synergy of any drug pair AB using the Bliss independence method described previously [16]:

$$S=(r_{A0}/r_{00})(r_{0B}/r_{00})-(r_{AB}/r_{00}),$$

such that *r*_*A0*_ represents the growth rate at a given concentration of drug A in the absence of B, and vice versa for *r*_*0B*_. *r*_*00*_ is the growth rate of the no-drug control, and *r*_*AB*_ is the growth rate at any concentration in which drugs A and B are found together. The degree of synergy *S* was only calculated for drug combinations that had growth rates larger than 0. Positive values indicate synergism, whereas negative ones denote antagonism.

### Drug combinations and interaction profile

To classify the interaction between 2 drugs, we considered an environment in which each drug separately inhibits 75% ± 10% of bacterial growth (IC75). For each combination, we evaluated 11 treatments: 9 different proportions of a given pair of antibiotics, a control of uninhibited growth, and a control with only M9 medium. Nine replicates for all treatments were considered, except for the M9 control that consisted of only 6 wells. This resulted in 81 replicates per drug combination and 4,212 for all 52 antibiotic pairs. OD measurements were taken every 15 min for 12 h, resulting in a total of 48 data points per individual replicate and 202,176 for all combinations and replicates.

To determine whether interactions were antagonistic, synergistic, or additive, we used a *t* test on the second-order term (α) of a quadratic regression of our data, as established previously [17]. The α parameter expresses convexity or concavity of observed bacterial-density data in the model *q(θ) = αθ*^*2*^ *+ βθ + γ*, such that θ represents any drug proportion between any drugs A and B (Fig 1B). Positive values of α indicate synergy and negative values antagonism.

### Collateral sensitivity network

We considered our previously published data on the evolved collateral effects of highly resistant populations of *P*. *aeruginosa* PA14 [30] and used the frequency of cross-resistance in all possible pairwise combinations of 8 of the drugs considered in this study. Briefly, the FCR counts the number of populations resistant to drug A that show collateral resistance to drug B, and vice versa, relative to the total number of populations resistant to A and B. Values close to 0 indicate reciprocal collateral sensitivity, and those close to 1 denote cross-resistance. We categorized the obtained values into 4 different groups and built a collateral sensitivity network (Fig 2): complete collateral sensitivity (FCR ≤ 0.25), partial collateral sensitivity (0.25 < FCR ≤ 0.5), partial cross-resistance (0.5 < FCR < 0.75), and complete cross-resistance (FCR ≥ 0.75).

### Experimental evolution of antibiotic combinations

Based on the interaction profile and the collateral sensitivity and/or resistance [30] scores, we selected a total of 38 different combinations for a series of evolution experiments (Fig 3A). For all combinations, we included 5 different proportions of the combined antibiotics, an uninhibited control, and an M9 control, resulting in 44 populations per combination, randomly distributed in a 96-well plate (2 combinations were included in a single plate), for a total of 1,672 populations. The concentration was set for each individual drug to inhibit bacterial growth by 75% (IC75). We considered 10 transfers (hereafter referred to as seasons) of 1% volume into fresh plates every 12 h (approximately 120 generations). For each season, OD_600_ measurements were taken every 15 min, resulting in 48 measurements per replicate and season and a total of 781,440 measurements across all replicate populations. All plates were frozen at −80 **°**C with 1:4 (v/v) of 86% glycerol.

To validate our OD measurements as a proxy for bacterial growth during evolution, we replicated the conditions of the first season for 4 selected combinations (only the 1:1 proportion), 6 corresponding single-drug treatments, and a no-drug control. We focused on those combinations and the corresponding monotherapies for which we also evaluated the influence of initial drug inhibitory level (Fig 7) and the evolution of resistance (S5 Fig). Each treatment was replicated 8 times. After 12 h of evolution, we performed a dilution series and standard plating techniques to count viable colony-forming units (CFUs) for all replicates and treatments. The obtained CFUs were then correlated with the endpoint OD measurements (S4 Fig). We found a significant correlation between our OD measurements and the CFU counts at the end of season 1 (Spearman rank correlation test, ρ_s_ = 0.782, *P* < 0.001). To further validate the OD measurements, we performed a similar correlation analysis for the same combinations and corresponding monotherapies, using evolved bacteria from the final transfer of the separate, focused evolution experiment, in which the influence of initial drug inhibitory levels was assessed. The evolved material was thawed from the frozen stock cultures, then exposed to 1 full season of experimental evolution under the exact treatment conditions already experienced by populations during the evolution experiment. Thereafter, CFUs were counted using a dilution series on Agar plates, as outlined above, and then compared to the OD measures obtained during the above repetition of a full season. As before, CFUs were significantly correlated with the corresponding OD measurements (Spearman rank correlation test, ρ_s_ = 0.339, *P =* 0.002).

We further validated the suitability of changes in growth characteristics as a proxy for evolutionary adaptation and therefore genetically fixed alterations by re-assessing cryo-preserved material from the last transfer of experimental evolution. This analysis was performed with material from the separate evolution experiment, which tested the influence of initial inhibitory levels, and further details are outlined below in the description of this experiment.

### Rates of adaptation

We first calculated the growth rate *r* as described above for each evolving population, treatment, and season. Subsequently, we considered the rate of adaptation for each evolving line as defined previously [16]:

$$R_{adapt}=\frac{\Delta r}{2\times t_{adapt}},$$

such that Δ*r* represents the change in growth rate over 10 seasons of growth, and the time of adaptation, *t*_adapt_, corresponds to the interpolated time at which a population reached half of its maximum growth rate. This measurement reflects how quickly resistance spreads in a population in a serial transfer experiment.

To determine to what extent adaptation to the drug combinations was determined by adaptation to each of the individual drugs, we measured which of the individual components in a drug pair led to lower and higher rates of adaptation. The single antibiotic in a pair that alone led to lower rates of adaptation was considered as the stronger of the components and the other as the weaker one. The adaptation rate of each combination was then standardized by the adaptation rate of either its weaker or stronger component drug. The 2 types of standardized adaptation rates were visualized in ACE networks and statistically evaluated (see below).

### BN analysis

We used BN analysis to assess the directional relationship between 4 variables, including the inferred drug interaction type, the frequency of collateral sensitivities, the adaptation rates, and the frequency of population extinctions. The entire BN analysis was repeated with the different types of inferred adaptation rates, including those obtained for the combinations in the main experiment and then those that we standardized by either the stronger or the weaker component drug.

The BN analysis generally followed 2 steps. In the first step, the approach identifies variables that are related to each other and visualizes these as nodes in a network between variables. In this step, it further infers the direction of each relationship and represents these as arrows in the network, thereby implying a causality between the connected variables [31]. To achieve this first step, the model first infers the graphical structure of the network by analyzing the probabilistic relations between all nodes and thereafter constructs the network by setting directions for the identified connections while satisfying an acyclicity constraint [58]. We implemented BN analysis employing a constraint-based interleaved incremental association–optimized algorithm [59] to reduce the likelihood of obtaining false positives and to obtain possible probabilistic dependencies between our variables: drug interaction type (categorical: synergism, additivity, or antagonism), FCR (categorical: complete collateral sensitivity, partial collateral sensitivity, partial cross-resistance, and complete cross-resistance), proportion of extinction (numerical), and rates of adaptation (numerical). We only included combinations with complete sets of data and then followed the algorithm’s default parameters. From the obtained dependencies, we estimated the conditional probabilities associated with the linked variables over an array of different values. All tests were performed in R using the “bnlearn” package [60].

### Additional correlation and partial correlation analysis

To validate the inferred dependencies from the BN analysis, we additionally performed correlation analysis combined with permutation tests, following the approach previously established for a similar analysis of ACE in *E*. *coli* [16]. For each round of permutation, we calculated correlation coefficients, ρ_s_, between any two given variables x and y by permuting the values of x while keeping y constant, as in [16]. For each test, we considered 10,000 permutations and estimated the *P* value as the proportion of the obtained distribution of correlation coefficients that had an absolute value larger than the absolute value obtained for the observed ρ_s_ [16]. This approach was used to correlate the measures of collateral effects and drug interaction to proportion of extinction and, later on, to the standardized adaptation rates.

Furthermore, to account for the effect of adaptation to the single drugs (z) in the main analysis with nonstandardized adaptation rates, we performed a partial correlation analysis with z as a covariate, generally following the previously established approach [16]. For this, we first obtained the residuals from the linear regression of x on z and those of y on z, such that y corresponds to the adaptation rates of the combination. Then, to estimate the correlation coefficient between x and y, with z as a covariate, we employed the permutation test as explained above using the residuals of the corresponding regressions [16].

### Experimental evolution with fixed inhibitory levels of antibiotic combinations

To evaluate the effect of the starting inhibition level of the combinations, we considered a second round of evolution experiments as described above. This time, the level of inhibition of the combination was fixed instead of that of the individual drug treatments. Briefly, concentrations of each drug were mixed 1:1 so that each would inhibit between 50% and 75% of growth. These were then diluted to obtain a range of different inhibition levels and to evaluate their effect on growth in *P*. *aeruginosa* after 12 h of incubation at 37 °C. Evolution experiments were then initiated for 4 different combinations that included 11 different treatments: a no-drug control, the individual monotherapies, and 8 different inhibition levels ranging from approximately IC50 to >IC90 of each combination. Each treatment was replicated 8 times and distributed randomly in 96-well plates.

### Genetically fixed changes in growth characteristics

We used the focused set-up of the above separate evolution experiment to validate the suitability of growth measurements as a proxy for evolutionary adaptation. Evolutionary adaptation assumes that changes are genetically fixed rather than due to phenotypic (i.e., physiological) responses. To assess this, we studied cryo-preserved material from the last drug-free season of the evolution experiment and regrew them under defined antibiotic conditions. Purely phenotypic adaptations to antibiotics are unlikely to have persisted for this material, which was grown under antibiotic-free conditions for 12 to 16 h (equivalent to a minimum of 6 generations) and additionally subjected to a cryo-preservation step. Therefore, any persistent changes in growth characteristics under antibiotic exposure are likely based on genetic changes and thus indicate evolutionary adaptation.

For this analysis, we considered material evolved in the presence of 2 synergistic (i.e., GEN plus CAR and STR plus PIT) or 2 antagonistic combinations (i.e, CIP plus GEN and CIP plus TOB), in all cases set to either IC50 or >IC90, and also included material from the corresponding monotherapies. A total of 4 replicate populations was studied for each of the various evolution treatments and compared to the ancestral PA14. Changes in growth characteristics were inferred from dose-response curves in a 2-fold dilution series of each of the antibiotics included in the pair. The evolved relative changes in resistance were calculated as the area under the curve (AUC) of the dose-response curve for each of the populations and then divided by that of the ancestral PA14. The results are shown in S5 Fig. They highlight a general increase in growth characteristics and thus resistance across the various treatment groups even if not significant in all cases (based on a 1-sample Wilcoxon test with μ = 1). We conclude that, overall, the observed changes in growth characteristics have a genetic basis and are not exclusively due to phenotypic responses. Therefore, we consider the recorded changes in growth characteristics to provide a meaningful proxy for evolutionary adaptation.

## Supporting information

S1 Fig Dose-response curves of the ancestral strain PA14 exposed to all different antibiotics used in the study. Each panel corresponds to a single antibiotic (see Table 1 for abbreviations). Boxplots show bacterial growth relative (*n* = 6 per concentration) to an antibiotic-free environment across different drug concentrations. The red dotted line indicates the 75% level of inhibition (IC75) used as a standard for subsequent experiments.
(TIF)

S2 Fig Validation of the interaction strength measure α. (A) Checkerboards of 8 selected combinations. Each panel corresponds to an antibiotic combination, here from left to right and top to bottom: CAR plus GEN, CAR plus CEF, STR plus PIT, TIC plus TOB, CIP plus CAR, CIP plus CEF, CIP plus DOR, and PIT plus CAR. Growth relative to the drug-free environment is shown over a grid of concentrations of both drugs in different shades of grey: values close to 1 indicate normal growth (black), whereas those close to 0 correspond to no detectable growth after 12 h of incubation (white). Red, grey, and blue circles embedded within each panel highlight the different types of interactions determined using α, showing—respectively—synergism, additivity, and antagonism. We calculated the degree of synergy (*S*) using the Bliss independence method either by averaging all obtained values across the grid where the fitness effect was measurable (panel B), or by calculating *S* from the combination having the same level of inhibition for each drug (panel C). A significant correlation was obtained between the degree of synergy *S* obtained in panels B and C with our measurements of α (as in S2 Fig). The data used for these panels are provided in S2 Data.
(TIF)

S3 Fig Interaction profile of 52 antibiotic combinations. Each panel shows the growth of the *P*. *aeruginosa* PA14 strain across 9 different drug proportions ranging from full dose of one drug (θ = 0) to a full dose of the second one (θ = 1), each set to inhibit 75% of normal growth. Points and error bars indicate the mean and 95% CI for bacterial growth, as inferred through OD (*n* = 9) after 12 h of incubation. Colored lines represent the quadratic fit of observed data, whereby the color itself indicates the interaction type, as deduced from the α parameter of the model. Synergy, additivity, and antagonism are shown as red, grey, or blue lines, respectively. The data used for these panels are provided in S3 Data. OD, optical density.
(TIF)

S4 Fig Validation of OD as a proxy for bacterial growth in the presence of antibiotics. We used the ancestral PA14 strain to replicate the first season of evolution for 6 selected single-drug treatments, 4 corresponding antibiotic combinations, and a no-drug control (shown in the different colors) in a single 96-well plate. Antibiotic concentrations were set to IC75 for the single-drug treatments, and for the drug pairs, each antibiotic was set to IC75 and then combined in a 1:1 ratio, thereby following the same set-up used for the main evolution experiment. For each treatment, we included 8 replicates. The plate was incubated at 37 °C for 12 h under continuous shaking. At the end of the incubation period, a sample from each well was taken, plated on LB agar plates, and incubated for 16 to 20 h at 37 °C to count the number of viable cells as CFUs. We found a significant correlation between the obtained CFU counts and the endpoint OD measurements (Spearman rank test, ρ_s_ = 0.782, *P* < 0.001). CFU, colony-forming unit; IC75, inhibiting 75% of bacterial growth; LB, Luria-Bertani; OD, optical density.
(TIF)

S5 Fig Changes in resistance upon experimental evolution in selected populations. We determined changes in resistance for selected populations from the separate evolution experiment with different initial drug inhibitory levels (Fig 7) that included 4 different combinations: (A) GEN plus CAR, (B) STR plus PIT, (C) CIP plus GEN, and (D) TOB plus CIP. For each of these combinations, we tested 4 populations adapted to each of the single drugs, 4 populations adapted to the combinations set to IC50, 4 populations adapted to those set to >IC90, and the ancestor PA14. All populations were from the final season with antibiotics. Antibiotic resistance was assessed with dose-response curves using 2-fold dilution series of each of the antibiotics included in the tested combination. Results are given as changes in resistance (for the changes in IC90, see S2 Table), calculated as the AUC for each evolved population relative to that of the ancestral PA14. Asterisks indicate significant differences obtained from a 1-sample Wilcoxon test (μ = 1, dotted red line). All *P* values were corrected for multiple comparison using FDR. AUC, area under the curve; FDR, false discovery rate; IC50, concentration inhibiting 50% of bacterial growth; IC90, concentration inhibiting 90% of bacterial growth.
(TIF)

S6 Fig Growth rate of the 50:50 treatment of all combinations over 10 seasons of experimental evolution. Each panel corresponds to an antibiotic combination, as indicated by the abbreviations in the top of each panel. Grey lines and circles show the growth rate *r* of each replicate within the 50:50 proportion. Orange circles and lines highlight the mean of all surviving populations per combination (note the number of replicate populations varies between combinations because of extinction; GEN plus CAR has no surviving population, and therefore it is not shown). The data used for these panels are provided in S4 Data.
(TIF)

S7 Fig Growth rates of the monotherapies over 10 seasons of experimental evolution. Each panel corresponds to a single-drug treatment. Grey lines and circles show the growth rate *r* of each replicate within a single-drug treatment (note that replicates among antibiotics differ; shown in brackets). Dark cyan circles and lines highlight the mean of all surviving populations per antibiotic. The data used for these panels are provided in S4 Data.
(TIF)

S8 Fig ACE networks for each drug interaction type. ACE networks were calculated for only synergistic (top panels), antagonistic (middle panels), and additive combinations (bottom panels). As in the main text, they were built on 2 parameters: (A, C, E) rates of adaptation and (B, D, F) extinction rates. Rates of adaptation and extinction numbers were calculated from the data provided in S4 Data. Abbreviations indicate antibiotics. A, azlocillin; ACE, antibiotic combination efficacy; C, ciprofloxacin; D, doripenem; F, cefsulodin; G, gentamicin; I, imipenem; K, carbenicillin; P, piperacillin + tazobactam; Q, ticarcillin; S, streptomycin; T, tobramycin; Z, ceftazidime.
(TIF)

S9 Fig ACE networks for the different collateral effects. ACE networks were calculated for only collaterally sensitive drug pairs (top panels) or collaterally resistant combinations (bottom panels). As in the main text, they were built on 2 parameters: (A, C) rates of adaptation and (B, D) extinction rates. Rates of adaptation and extinction numbers were calculated from the data provided in S4 Data. Abbreviations indicate antibiotics. A, azlocillin; ACE, antibiotic combination efficacy; C, ciprofloxacin; D, doripenem; F, cefsulodin; G, gentamicin; I, imipenem; K, carbenicillin; P, piperacillin + tazobactam; Q, ticarcillin; S, streptomycin; T, tobramycin; Z, ceftazidime.
(TIF)

S10 Fig Rates of adaptation in single-drug treatments. Rates of adaptation are shown for the treatments with only 1 antibiotic. Colors indicate the different antibiotic classes: fluoroquinolones (red), carbapenems (green), cephalosporins (gold), penicillins (orange), and aminoglycosides (light blue). The number of populations in each treatment varies depending on the number of times a given drug is part of the tested combinations and the number of extinct populations (shown in brackets for each drug). The data used for these panels are provided in S4 Data.
(TIF)

S11 Fig Changes in growth rate of 4 selected combinations with fixed initial inhibitory levels. Each column corresponds to a specific antibiotic combination, and the rows represent the different initial inhibitory levels considered: from top to bottom are shown the no-drug controls (black), the monotherapies (turquoise and purple; the numbers given in brackets after the antibiotic abbreviation indicates which monotherapy is shown first or second), and 8 different starting levels of inhibition of the combinations (from approximately IC50 in yellow to >IC95 in dark red). Grey points and lines indicate the replicate population, while the colored points and lines show the mean per treatment and combination. Rates of adaptation, extinction numbers, and inhibitory levels were calculated from the data provided in S5 Data. IC50, concentration inhibiting 50% of bacterial growth; IC90, concentration inhibiting 90% of bacterial growth.
(TIF)

S1 Table The α test of 52 drug combinations used against *P*. *aeruginosa*. (DOCX)

S2 Table Rates of adaptation of all combinations relative to the weaker and stronger components in a drug pair. (DOCX)

S3 Table Effect test of the initial inhibitory level, interaction type, and combination on rates of adaptation. (DOCX)

S4 Table Effect test of the initial inhibitory level, interaction type, and combination on the number of extinctions. (DOCX)

S1 Data Key to datasets (Readme file). (RTF)

S2 Data OD measurements after 12 h of growth in different proportions of a given drug pair for all 52 antibiotic combinations. These data were used to infer the drug interaction type using the α estimator.
(TXT)

S3 Data OD measurements after 12 h of growth in drug checkerboards evaluating the interaction of 8 selected antibiotic combinations. These data were used to calculate the degree of synergy *S*, to correlate it to the values obtained for the same drug pairs using the α estimator.
(TXT)

S4 Data OD measurements taken every 15 min for 38 antibiotic pairs during a total of 120 h. These data were then used to infer adaptation rates in surviving replicate populations and the number of extinction events occurring per combination and treatment.
(TXT)

S5 Data OD measurements taken every 15 min for 4 selected antibiotic pairs with varying levels of inhibition during a total of 120 h. These data were then used to infer adaptation rates in surviving replicate populations and the number of extinction events occurring per combination and treatment.
(TXT)

## Abbreviations

ACE: antibiotic combination efficacy
AUC: area under the curve
AUCi: area under the curve of relative inhibition of growth
AZL: azlocillin
BN: Bayesian network
CAR: carbenicillin
CEF: cefsulodin
CEZ: ceftazidime
CFU: colony-forming unit
CIP: ciprofloxacin
DOR: doripenem
FCR: frequency of collateral resistances
GEN: gentamicin
IC: inhibitory concentration
IC50: concentration inhibiting 50% of bacterial growth
IC75: concentration inhibiting 75% of bacterial growth
IC90: concentration inhibiting 90% of bacterial growth
IMI: imipenem
MIC: minimum inhibitory concentration
OD: optical density
PIT: piperacillin + tazobactam
STR: streptomycin
TIC: ticarcillin
TOB: tobramycin
