# Supplementary figures and images for: Antibiotic combination efficacy (ACE) networks for a Pseudomonas aeruginosa model
Source: PLoS Biol. 2018 Apr 30;16(4):e2004356. doi: 10.1371/journal.pbio.2004356 (PMC5945231; doi:10.1371/journal.pbio.2004356)

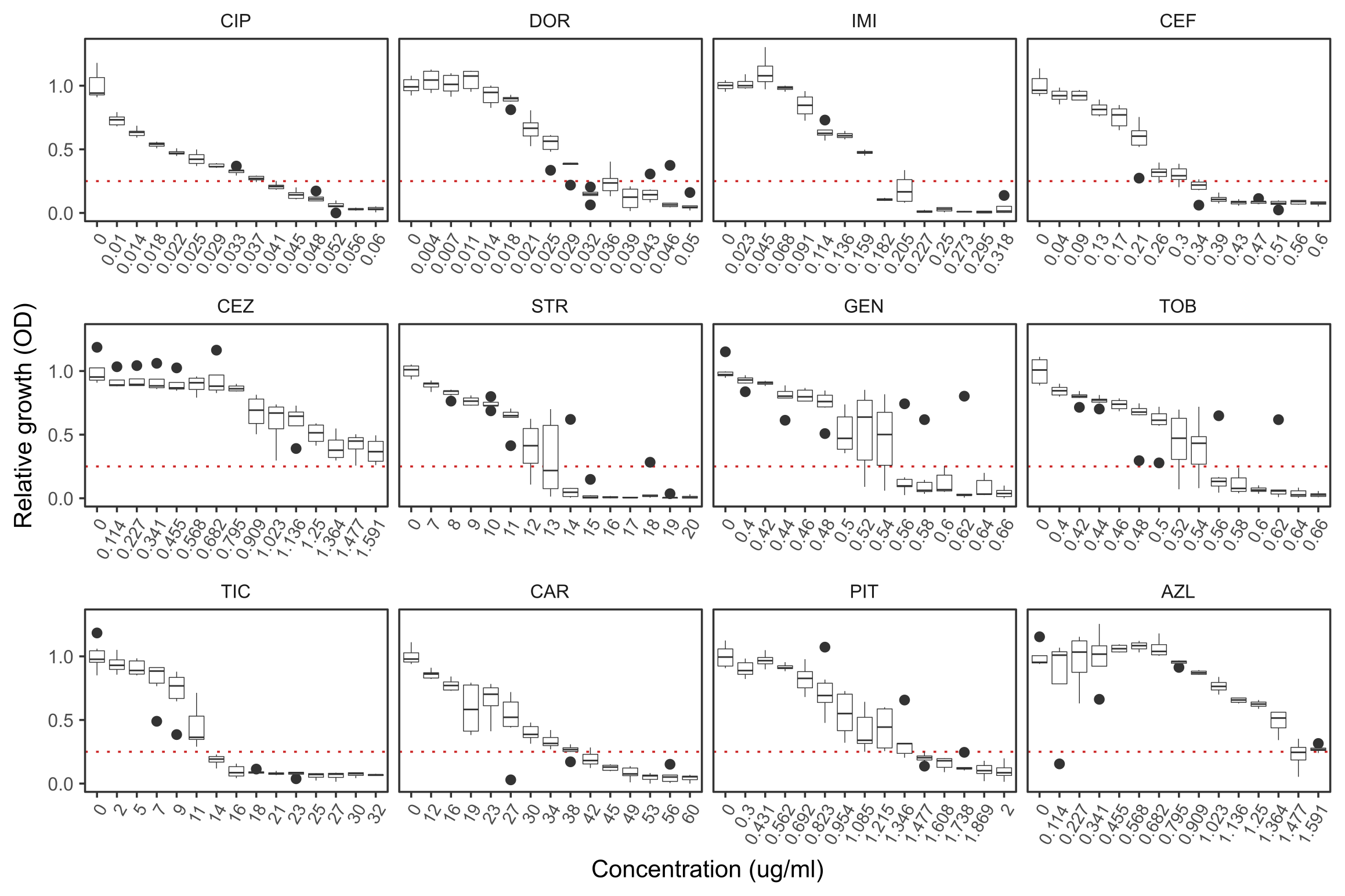

Supplement: S1 Fig — Each panel corresponds to a single antibiotic (see Table 1 for abbreviations). Boxplots show bacterial growth relative (n = 6 per concentration) to an antibiotic-free environment across different drug concentrations. The red dotted line indicates the 75% level of inhibition (IC75) used as a standard for subsequent experiments. (TIF) [file pbio.2004356.s001.tif]

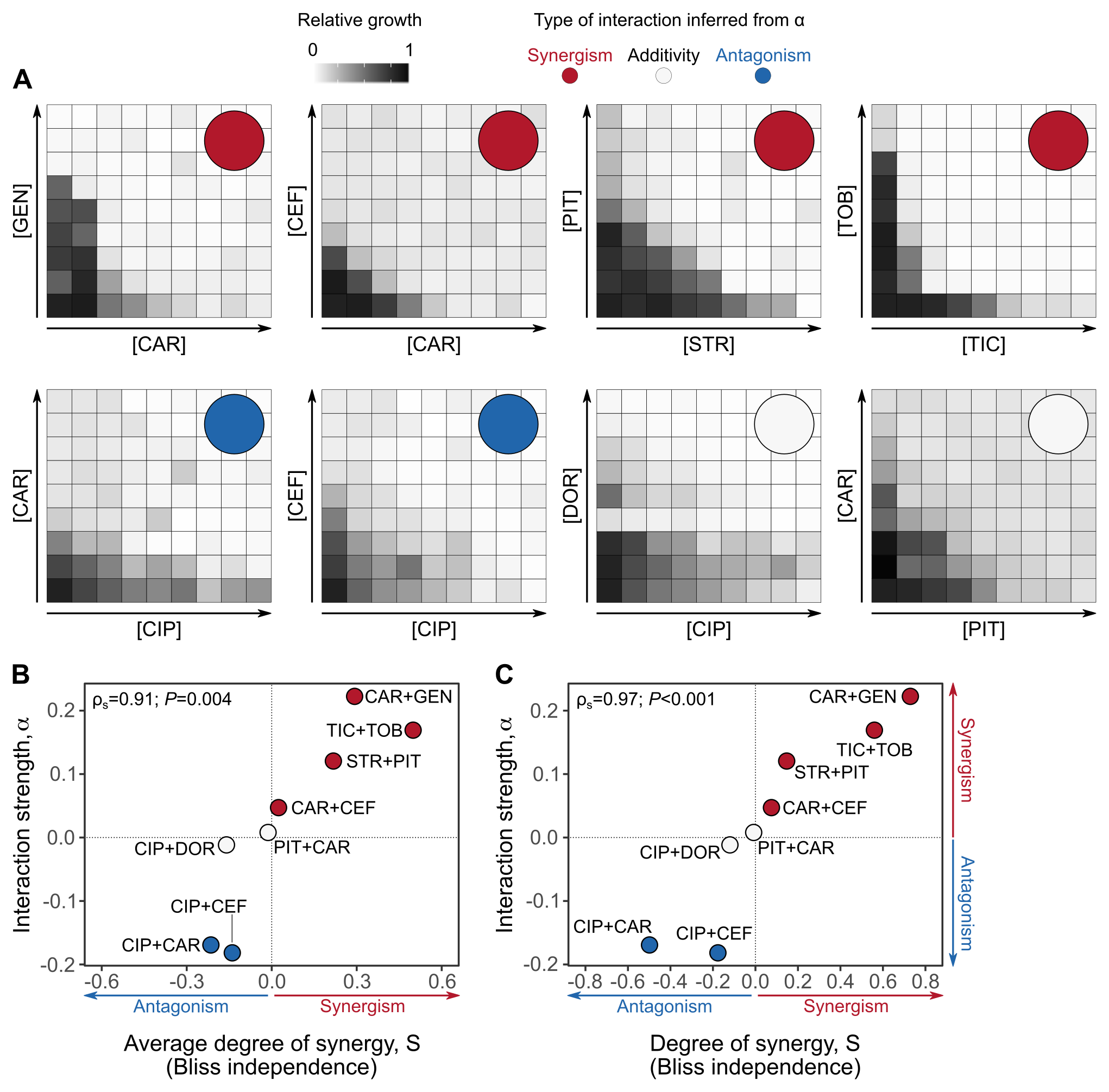

Supplement: S2 Fig — (A) Checkerboards of 8 selected combinations. Each panel corresponds to an antibiotic combination, here from left to right and top to bottom: CAR plus GEN, CAR plus CEF, STR plus PIT, TIC plus TOB, CIP plus CAR, CIP plus CEF, CIP plus DOR, and PIT plus CAR. Growth relative to the drug-free environment is shown over a grid of concentrations of both drugs in different shades of grey: values close to 1 indicate normal growth (black), whereas those close to 0 correspond to no detectable growth after 12 h of incubation (white). Red, grey, and blue circles embedded within each panel highlight the different types of interactions determined using α, showing—respectively—synergism, additivity, and antagonism. We calculated the degree of synergy (S) using the Bliss independence method either by averaging all obtained values across the grid where the fitness effect was measurable (panel B), or by calculating S from the combination having the same level of inhibition for each drug (panel C). A significant correlation was obtained between the degree of synergy S obtained in panels B and C with our measurements of α (as in S2 Fig). The data used for these panels are provided in S2 Data. (TIF) [file pbio.2004356.s002.tif]

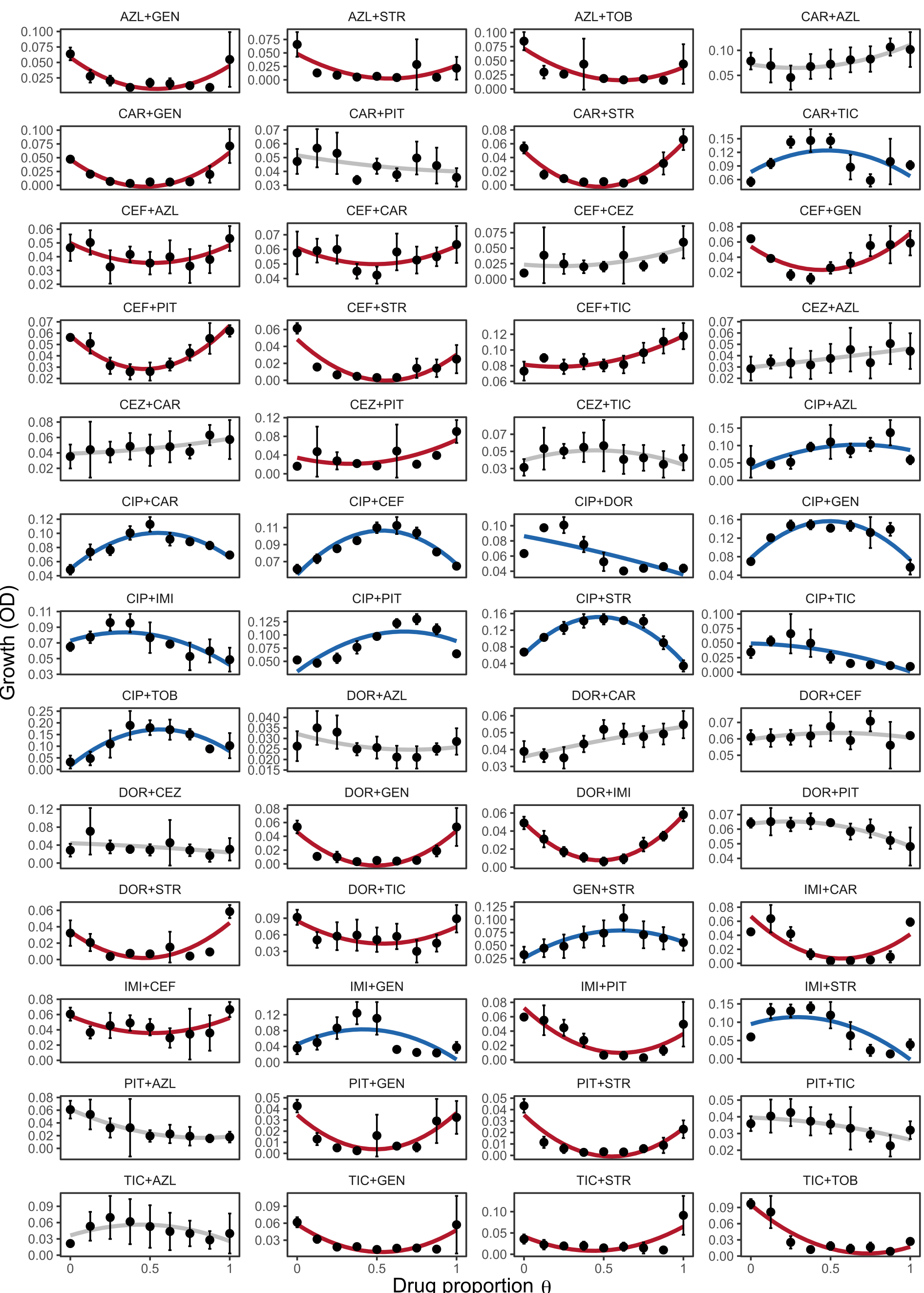

Supplement: S3 Fig — Each panel shows the growth of the P. aeruginosa PA14 strain across 9 different drug proportions ranging from full dose of one drug (θ = 0) to a full dose of the second one (θ = 1), each set to inhibit 75% of normal growth. Points and error bars indicate the mean and 95% CI for bacterial growth, as inferred through OD (n = 9) after 12 h of incubation. Colored lines represent the quadratic fit of observed data, whereby the color itself indicates the interaction type, as deduced from the α parameter of the model. Synergy, additivity, and antagonism are shown as red, grey, or blue lines, respectively. The data used for these panels are provided in S3 Data. OD, optical density. (TIF) [file pbio.2004356.s003.tif]

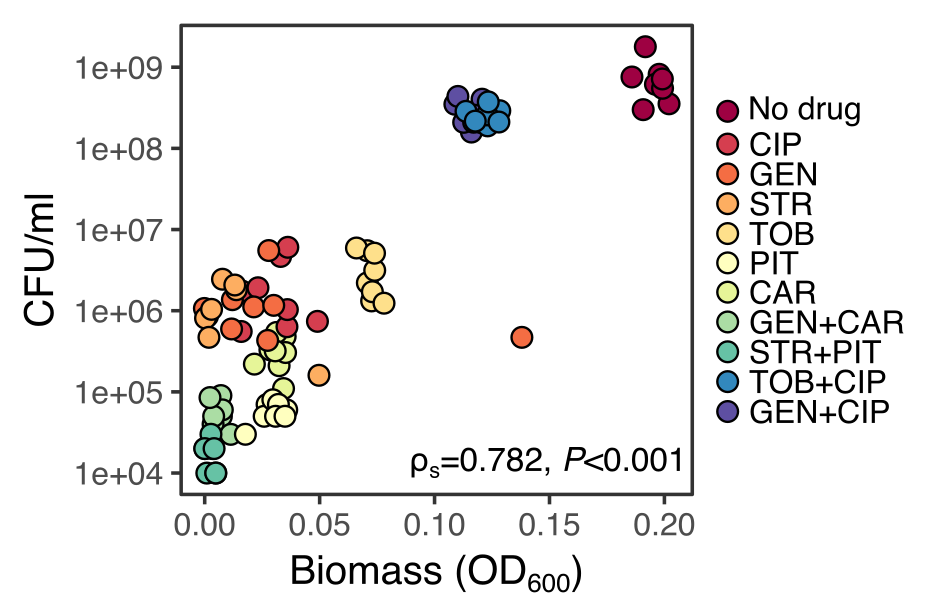

Supplement: S4 Fig — We used the ancestral PA14 strain to replicate the first season of evolution for 6 selected single-drug treatments, 4 corresponding antibiotic combinations, and a no-drug control (shown in the different colors) in a single 96-well plate. Antibiotic concentrations were set to IC75 for the single-drug treatments, and for the drug pairs, each antibiotic was set to IC75 and then combined in a 1:1 ratio, thereby following the same set-up used for the main evolution experiment. For each treatment, we included 8 replicates. The plate was incubated at 37 °C for 12 h under continuous shaking. At the end of the incubation period, a sample from each well was taken, plated on LB agar plates, and incubated for 16 to 20 h at 37 °C to count the number of viable cells as CFUs. We found a significant correlation between the obtained CFU counts and the endpoint OD measurements (Spearman rank test, ρs = 0.782, P < 0.001). CFU, colony-forming unit; IC75, inhibiting 75% of bacterial growth; LB, Luria-Bertani; OD, optical density. (TIF) [file pbio.2004356.s004.tif]

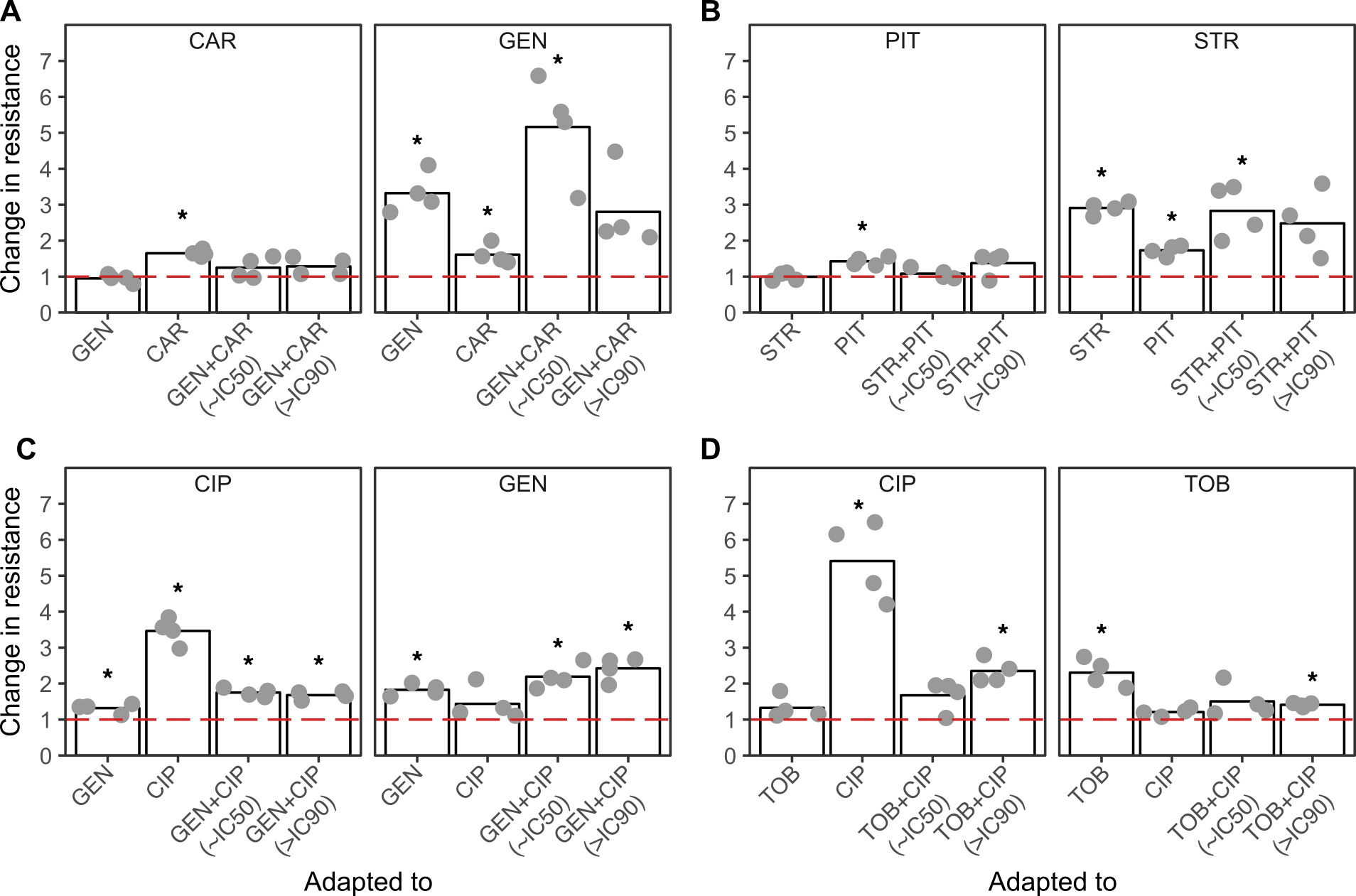

Supplement: S5 Fig — We determined changes in resistance for selected populations from the separate evolution experiment with different initial drug inhibitory levels (Fig 7) that included 4 different combinations: (A) GEN plus CAR, (B) STR plus PIT, (C) CIP plus GEN, and (D) TOB plus CIP. For each of these combinations, we tested 4 populations adapted to each of the single drugs, 4 populations adapted to the combinations set to IC50, 4 populations adapted to those set to >IC90, and the ancestor PA14. All populations were from the final season with antibiotics. Antibiotic resistance was assessed with dose-response curves using 2-fold dilution series of each of the antibiotics included in the tested combination. Results are given as changes in resistance (for the changes in IC90, see S2 Table), calculated as the AUC for each evolved population relative to that of the ancestral PA14. Asterisks indicate significant differences obtained from a 1-sample Wilcoxon test (μ = 1, dotted red line). All P values were corrected for multiple comparison using FDR. AUC, area under the curve; FDR, false discovery rate; IC50, concentration inhibiting 50% of bacterial growth; IC90, concentration inhibiting 90% of bacterial growth. (TIF) [file pbio.2004356.s005.tif]

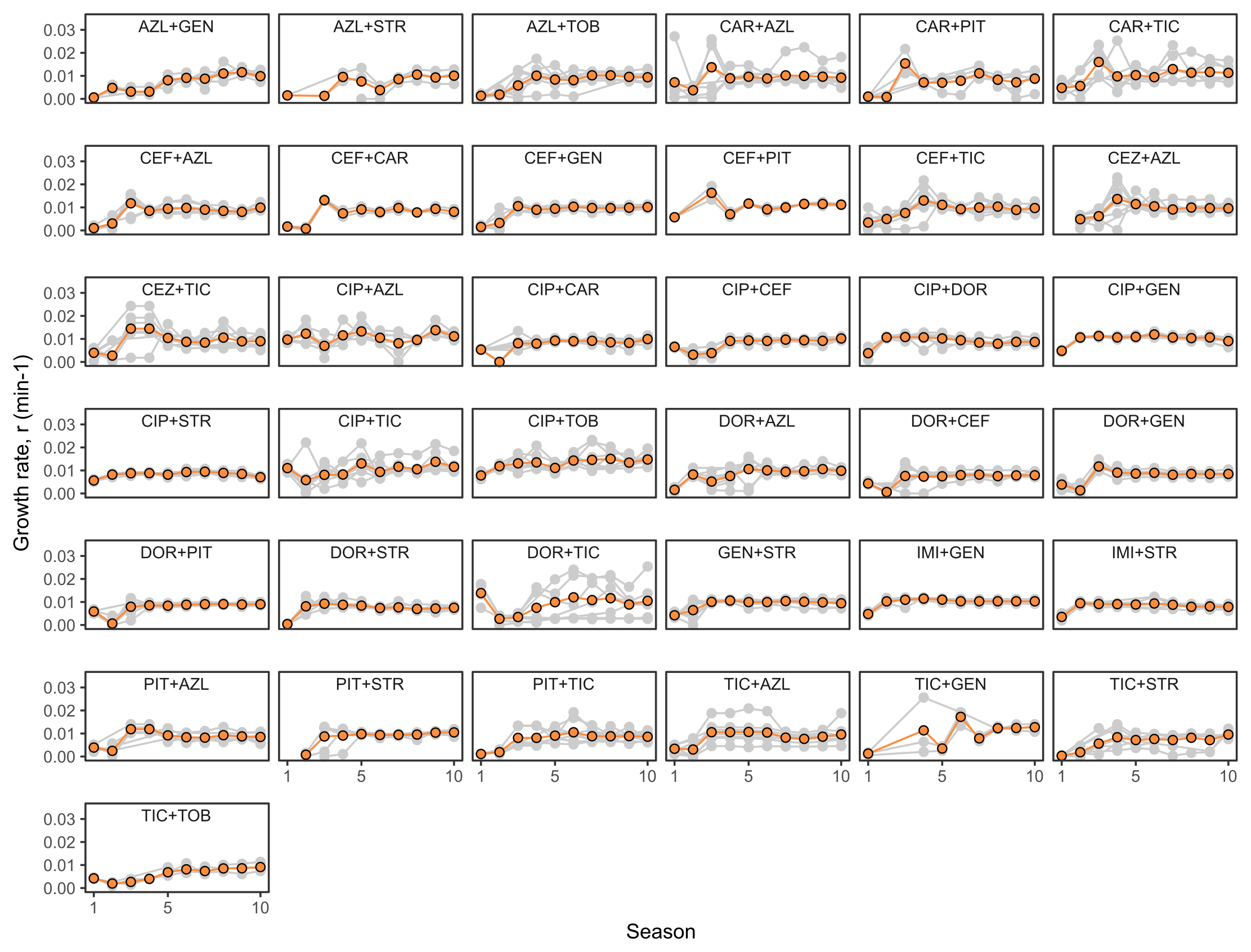

Supplement: S6 Fig — Each panel corresponds to an antibiotic combination, as indicated by the abbreviations in the top of each panel. Grey lines and circles show the growth rate r of each replicate within the 50:50 proportion. Orange circles and lines highlight the mean of all surviving populations per combination (note the number of replicate populations varies between combinations because of extinction; GEN plus CAR has no surviving population, and therefore it is not shown). The data used for these panels are provided in S4 Data. (TIF) [file pbio.2004356.s006.tif]

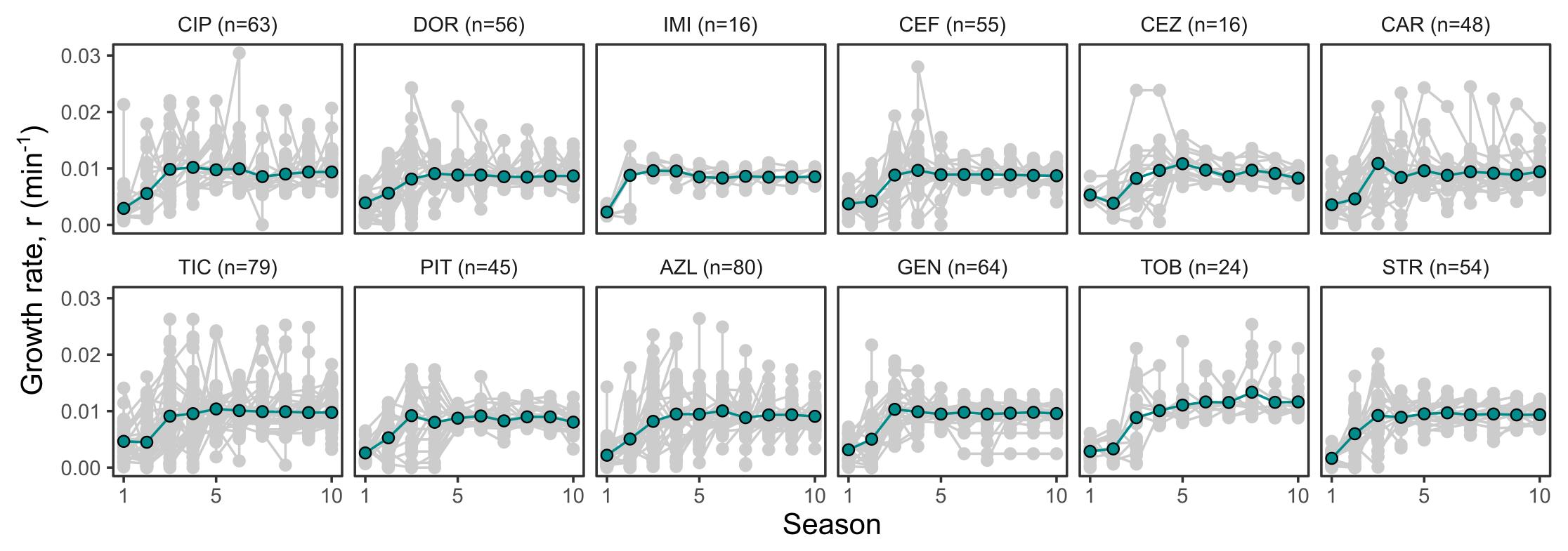

Supplement: S7 Fig — Each panel corresponds to a single-drug treatment. Grey lines and circles show the growth rate r of each replicate within a single-drug treatment (note that replicates among antibiotics differ; shown in brackets). Dark cyan circles and lines highlight the mean of all surviving populations per antibiotic. The data used for these panels are provided in S4 Data. (TIF) [file pbio.2004356.s007.tif]

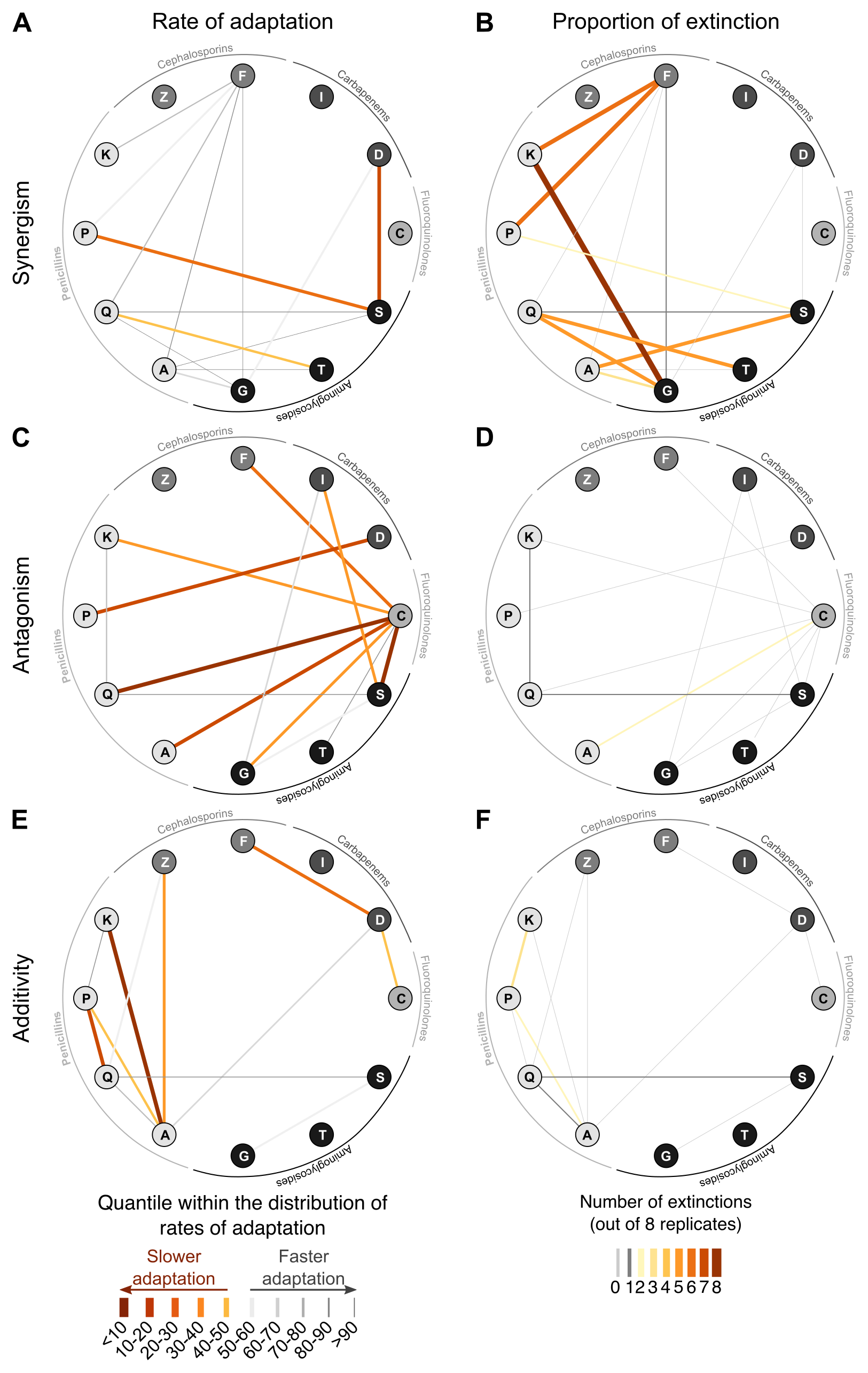

Supplement: S8 Fig — ACE networks were calculated for only synergistic (top panels), antagonistic (middle panels), and additive combinations (bottom panels). As in the main text, they were built on 2 parameters: (A, C, E) rates of adaptation and (B, D, F) extinction rates. Rates of adaptation and extinction numbers were calculated from the data provided in S4 Data. Abbreviations indicate antibiotics. A, azlocillin; ACE, antibiotic combination efficacy; C, ciprofloxacin; D, doripenem; F, cefsulodin; G, gentamicin; I, imipenem; K, carbenicillin; P, piperacillin + tazobactam; Q, ticarcillin; S, streptomycin; T, tobramycin; Z, ceftazidime. (TIF) [file pbio.2004356.s008.tif]

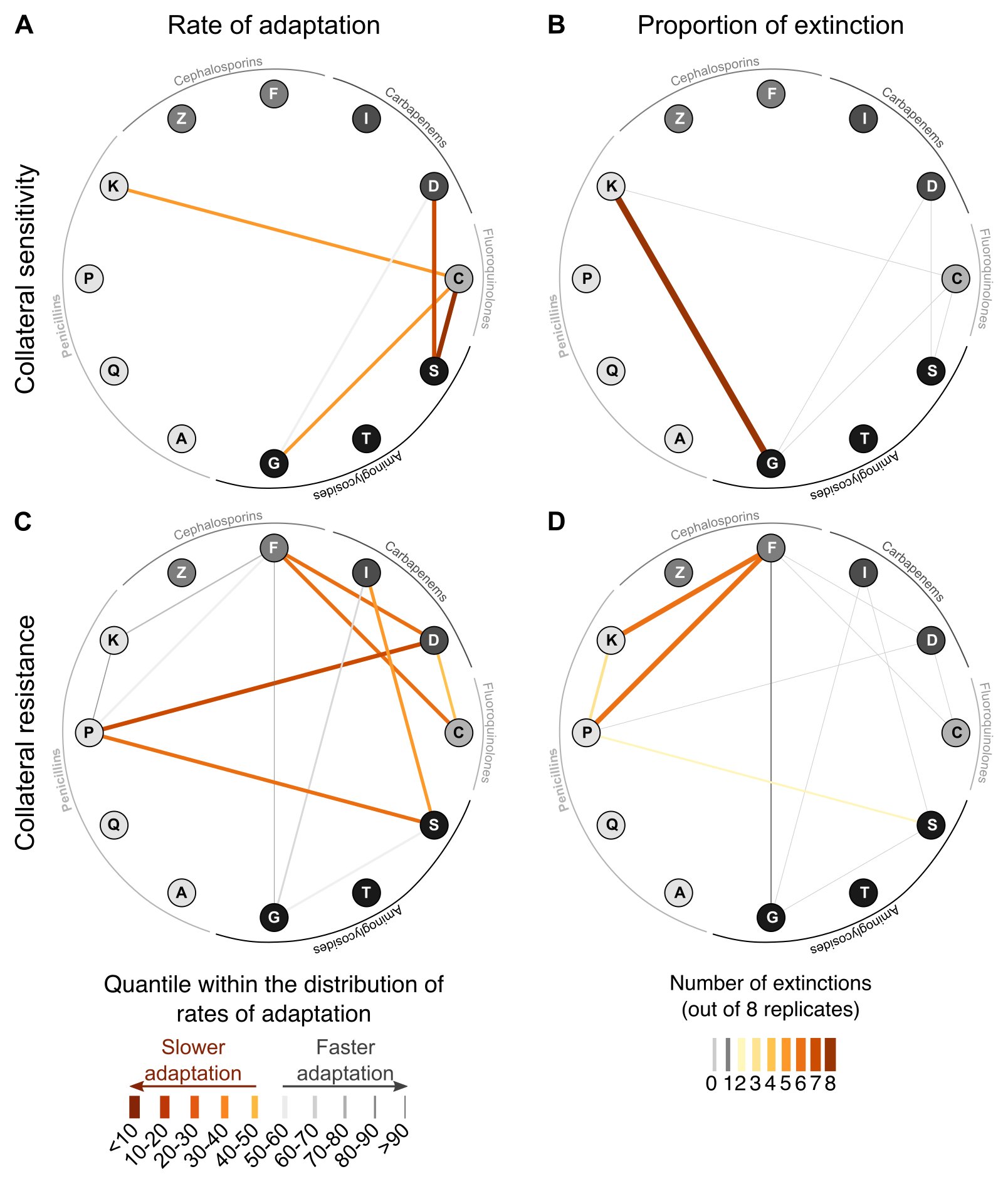

Supplement: S9 Fig — ACE networks were calculated for only collaterally sensitive drug pairs (top panels) or collaterally resistant combinations (bottom panels). As in the main text, they were built on 2 parameters: (A, C) rates of adaptation and (B, D) extinction rates. Rates of adaptation and extinction numbers were calculated from the data provided in S4 Data. Abbreviations indicate antibiotics. A, azlocillin; ACE, antibiotic combination efficacy; C, ciprofloxacin; D, doripenem; F, cefsulodin; G, gentamicin; I, imipenem; K, carbenicillin; P, piperacillin + tazobactam; Q, ticarcillin; S, streptomycin; T, tobramycin; Z, ceftazidime. (TIF) [file pbio.2004356.s009.tif]

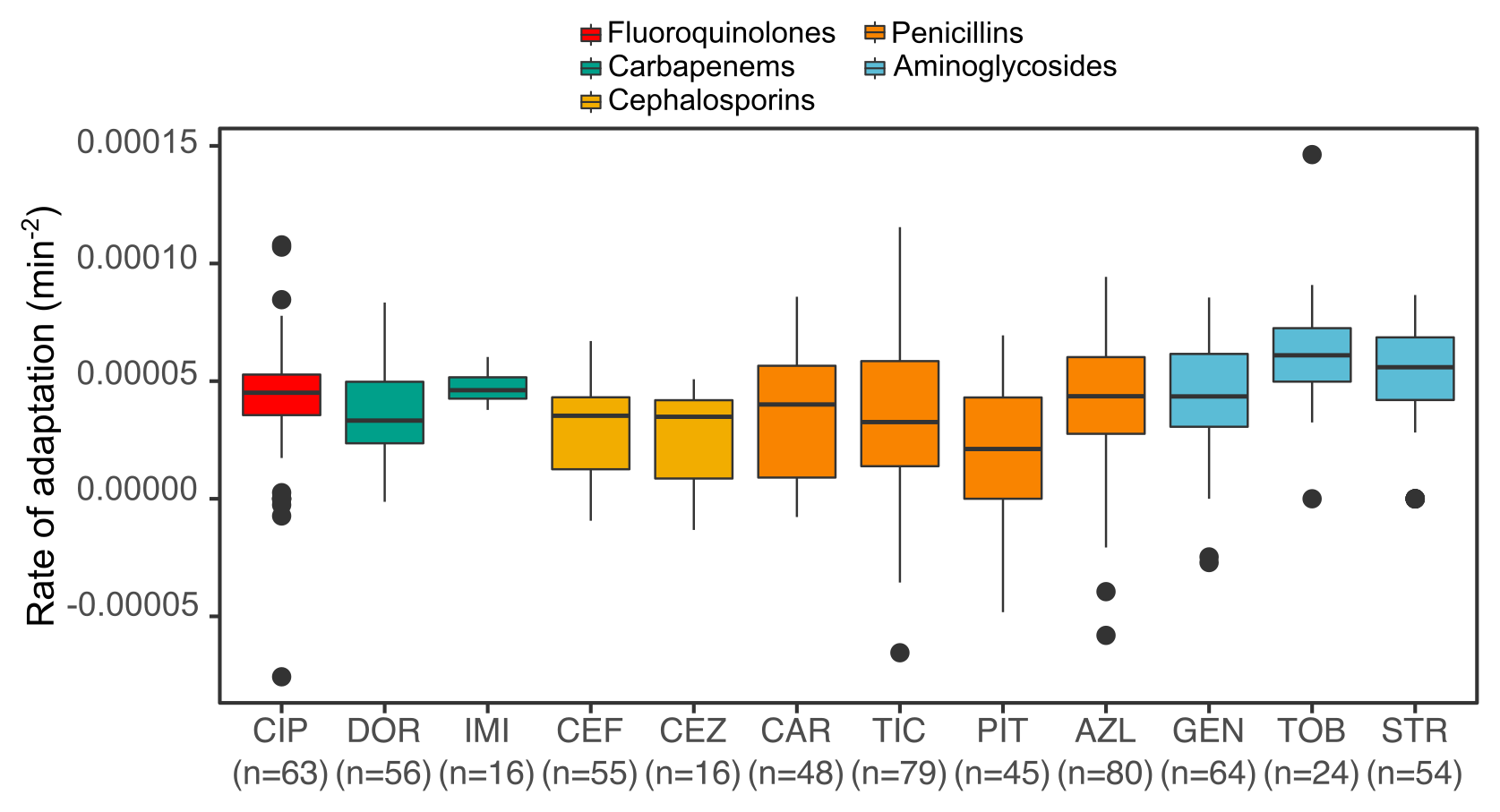

Supplement: S10 Fig — Rates of adaptation are shown for the treatments with only 1 antibiotic. Colors indicate the different antibiotic classes: fluoroquinolones (red), carbapenems (green), cephalosporins (gold), penicillins (orange), and aminoglycosides (light blue). The number of populations in each treatment varies depending on the number of times a given drug is part of the tested combinations and the number of extinct populations (shown in brackets for each drug). The data used for these panels are provided in S4 Data. (TIF) [file pbio.2004356.s010.tif]

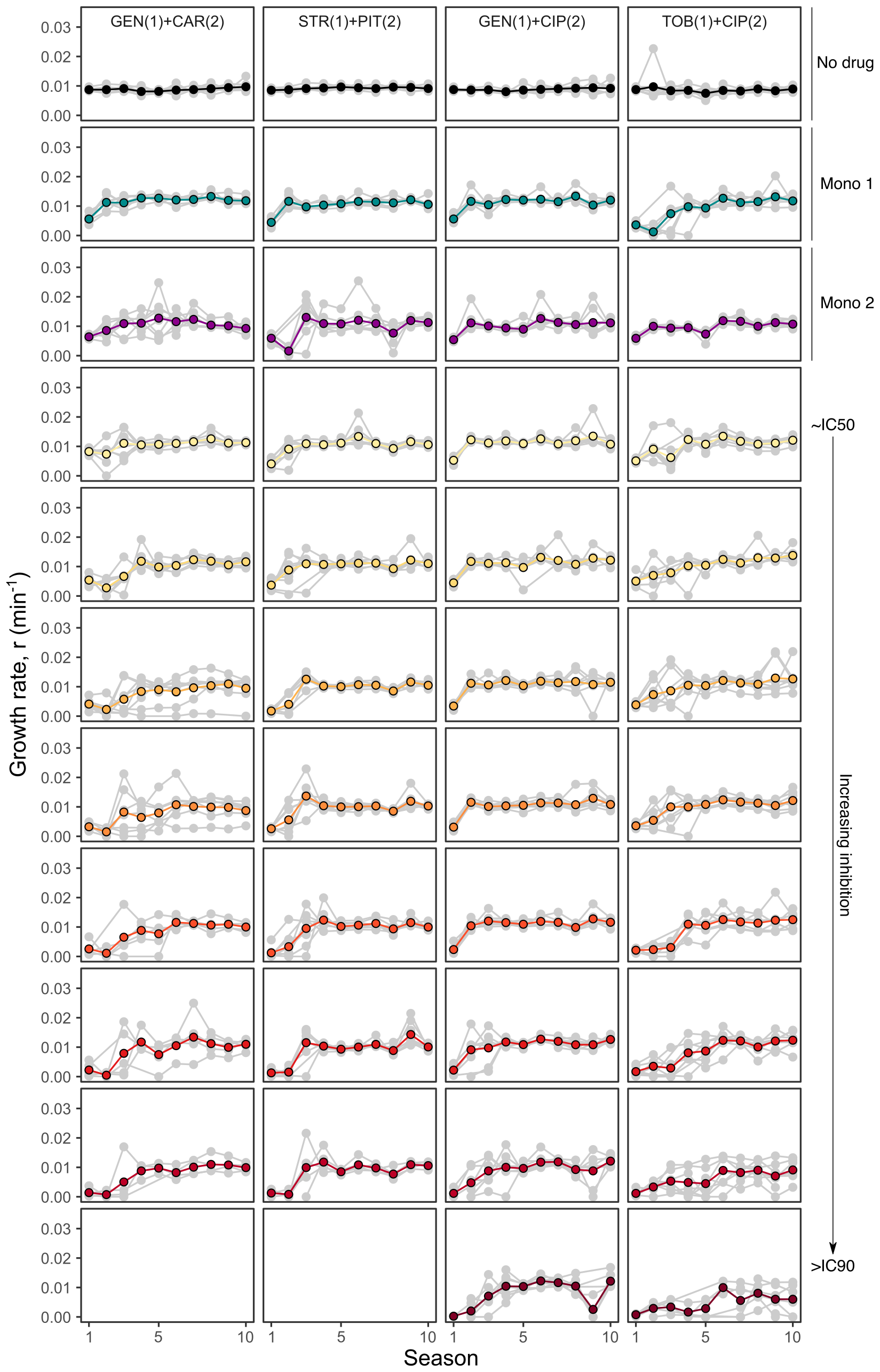

Supplement: S11 Fig — Each column corresponds to a specific antibiotic combination, and the rows represent the different initial inhibitory levels considered: from top to bottom are shown the no-drug controls (black), the monotherapies (turquoise and purple; the numbers given in brackets after the antibiotic abbreviation indicates which monotherapy is shown first or second), and 8 different starting levels of inhibition of the combinations (from approximately IC50 in yellow to >IC95 in dark red). Grey points and lines indicate the replicate population, while the colored points and lines show the mean per treatment and combination. Rates of adaptation, extinction numbers, and inhibitory levels were calculated from the data provided in S5 Data. IC50, concentration inhibiting 50% of bacterial growth; IC90, concentration inhibiting 90% of bacterial growth. (TIF) [file pbio.2004356.s011.tif]
