# Supplementary material for: Antibiotic combination efficacy (ACE) networks for a Pseudomonas aeruginosa model
Source: PLoS Biol. 2018 Apr 30;16(4):e2004356. doi: 10.1371/journal.pbio.2004356 (PMC5945231; doi:10.1371/journal.pbio.2004356)
Supplement: S1 Table — (DOCX) [file pbio.2004356.s012.docx]

**S1 Table. The alpha test of 52 drug combinations used against *P. aeruginosa*.**

| **Drug 1** | **Drug 2** | **Alpha** | ***P* (alpha)^a^** | **SE^b^** |
| --- | --- | --- | --- | --- |
| CAR | AZL | 0.088 | 0.066 | 0.047 |
| CEF | AZL | 0.055 | 0.004 | 0.018 |
| CEZ | AZL | 0.002 | 0.942 | 0.025 |
| CIP | AZL | -0.147 | 0.010 | 0.056 |
| DOR | AZL | 0.015 | 0.208 | 0.012 |
| AZL | GEN | 0.176 | 0.000 | 0.031 |
| PIT | AZL | 0.066 | 0.067 | 0.035 |
| AZL | STR | 0.137 | <0.001 | 0.036 |
| TIC | AZL | -0.100 | 0.083 | 0.057 |
| AZL | TOB | 0.151 | <0.001 | 0.039 |
| CEF | CAR | 0.047 | 0.012 | 0.018 |
| CIP | CAR | -0.169 | <0.001 | 0.016 |
| DOR | CAR | -0.004 | 0.737 | 0.011 |
| CAR | GEN | 0.222 | <0.001 | 0.023 |
| IMI | CAR | 0.186 | <0.001 | 0.023 |
| CAR | PIT | 0.008 | 0.680 | 0.019 |
| CAR | STR | 0.231 | <0.001 | 0.017 |
| CIP | CEF | -0.182 | <0.001 | 0.012 |
| DOR | CEF | -0.014 | 0.314 | 0.013 |
| CEF | GEN | 0.156 | <0.001 | 0.025 |
| IMI | CEF | 0.085 | 0.008 | 0.031 |
| CEF | PIT | 0.135 | <0.001 | 0.014 |
| CEF | STR | 0.157 | <0.001 | 0.017 |
| CIP | DOR | -0.012 | 0.605 | 0.022 |
| CIP | GEN | -0.327 | <0.001 | 0.031 |
| CIP | IMI | -0.094 | <0.001 | 0.025 |
| CIP | STR | -0.397 | <0.001 | 0.024 |
| DOR | GEN | 0.198 | <0.001 | 0.020 |
| DOR | IMI | 0.184 | <0.001 | 0.011 |
| IMI | GEN | -0.221 | <0.001 | 0.051 |
| CIP | PIT | -0.168 | <0.001 | 0.026 |
| DOR | PIT | -0.029 | 0.020 | 0.012 |
| PIT | GEN | 0.127 | <0.001 | 0.021 |
| IMI | PIT | 0.171 | <0.001 | 0.027 |
| PIT | STR | 0.121 | <0.001 | 0.010 |
| DOR | STR | 0.150 | <0.001 | 0.020 |
| IMI | STR | -0.229 | <0.001 | 0.052 |
| GEN | STR | -0.145 | <0.001 | 0.039 |
| CAR | TIC | -0.208 | <0.001 | 0.052 |
| CEF | TIC | 0.063 | 0.005 | 0.022 |
| CEZ | TIC | -0.056 | 0.089 | 0.033 |
| CIP | TIC | -0.043 | 0.170 | 0.031 |
| DOR | TIC | 0.143 | 0.001 | 0.041 |
| TIC | GEN | 0.175 | <0.001 | 0.032 |
| PIT | TIC | -0.010 | 0.490 | 0.014 |
| TIC | STR | 0.178 | <0.001 | 0.036 |
| TIC | TOB | 0.169 | <0.001 | 0.025 |
| CIP | TOB | -0.490 | <0.001 | 0.055 |
| CEZ | CAR | 0.012 | 0.740 | 0.036 |
| CEF | CEZ | 0.047 | 0.291 | 0.044 |
| DOR | CEZ | -0.008 | 0.868 | 0.051 |
| CEZ | PIT | 0.116 | 0.032 | 0.053 |

A quadratic model was fit to observed growth data after 12 h of incubation at 37˚C. The first parameter of the model (alpha) was used to determine significance of synergism or antagonism between any two drugs. Positive values of alpha indicate synergism whereas negative values represent antagonisms. Values of alpha not significantly different from 0 (*P* alpha) are considered as additive interactions.

^a^ *P*: P-values obtained from a one-sample t-student test (µ=0) of alpha.

^b^ SE: Standard error of the parameter alpha.
