# Supplementary material for: Antibiotic combination efficacy (ACE) networks for a Pseudomonas aeruginosa model
Source: PLoS Biol. 2018 Apr 30;16(4):e2004356. doi: 10.1371/journal.pbio.2004356 (PMC5945231; doi:10.1371/journal.pbio.2004356)
Supplement: S2 Table — (DOCX) [file pbio.2004356.s013.docx]

**S3 Table. Rates of adaptation of all combinations relative to the weaker and stronger components in a drug pair.**

| **Drug 1** | **Drug 2** | **Performance relative to the weaker component (%)** | **Performance relative to the stronger component (%)** | **Quantile of the rate of adaptation in the combinations (%)** |
| --- | --- | --- | --- | --- |
| AZL | CAR | 90.5 | 88.8 | <10 |
| AZL | CEF | 2.9 | 131.4 | 80-90 |
| AZL | CEZ | 39.6 | 10.8 | 30-40 |
| AZL | CIP | 82 | 76.7 | 10-20 |
| AZL | DOR | 21.2 | 13.2 | 60-70 |
| AZL | GEN | 25 | 21.1 | 60-70 |
| AZL | PIT | 14.7 | 89 | 40-50 |
| AZL | STR | 11.1 | 130.4 | >90 |
| AZL | TIC | 59 | 64.8 | 70-80 |
| AZL | TOB | 4.3 | 57 | >90 |
| CAR | CEF | 6.9 | 8.7 | 70-80 |
| CAR | PIT | 3.3 | 6.9 | 80-90 |
| CEF | DOR | 33.4 | 29.5 | 20-30 |
| CEF | PIT | 17.5 | 109.7 | 50-60 |
| CIP | CAR | 37.5 | 30 | 30-40 |
| CIP | CEF | 41.6 | 15.3 | 20-30 |
| CIP | DOR | 3.6 | 69.6 | 40-50 |
| CIP | TOB | 37.5 | 29 | 80-90 |
| CIP | GEN | 33.8 | 16 | 30-40 |
| DOR | GEN | 35.6 | 61.9 | 50-60 |
| DOR | PIT | 14 | 11.3 | 10-20 |
| GEN | CEF | 52.4 | 81.2 | >90 |
| GEN | IMI | 13 | 9 | 60-70 |
| GEN | STR | 20.9 | 2.6 | 50-60 |
| STR | CIP | 74.7 | 74.2 | <10 |
| STR | DOR | 70.4 | 61.8 | 10-20 |
| STR | IMI | 42.3 | 29.1 | 30-40 |
| STR | PIT | 57.7 | 42.3 | 20-30 |
| TIC | CAR | 8.7 | 20.1 | 70-80 |
| TIC | CEF | 15.6 | 18 | 70-80 |
| TIC | CEZ | 102.6 | 141.2 | 50-60 |
| TIC | CIP | 89.6 | 87.3 | <10 |
| TIC | GEN | 34.5 | 48.4 | >90 |
| TIC | PIT | 31.6 | 19.9 | 10-20 |
| TIC | DOR | 7652.3 | 81.95 |  |
| TIC | STR | 0.1 | 89.8 | 80-90 |
| TIC | TOB | 27.6 | 5.4 | 40-50 |

Performance of the combination represents how much slower (orange cells) or faster (grey cells) *P. aeruginosa* adapted in multi-drug environments relative to adaptation to the corresponding single drugs. The stronger component is defined as the drug within a drug pair that alone (i.e., in monotherapy) led to lower rates of adaptation (and thus more effective inhibition of adaptation) compared to the second drug. The performance (P) of each combination is then calculated by $P=100\times\left(1-\frac{r_{combination}}{r_{strong/weak}}\right)$. Positive values (orange) indicate that the adaptation to the combined drugs was faster (in percentage) than to the respective individual component. Negative values (grey) indicate that adaptation to the combination was slower than the corresponding single drug.
