# Supplementary material for: Antibiotic combination efficacy (ACE) networks for a Pseudomonas aeruginosa model
Source: PLoS Biol. 2018 Apr 30;16(4):e2004356. doi: 10.1371/journal.pbio.2004356 (PMC5945231; doi:10.1371/journal.pbio.2004356)
Supplement: S3 Table — (DOCX) [file pbio.2004356.s014.docx]

**S4 Table. Effect test of the initial inhibitory level, interaction type, and combination on rates of adaptation.**

| **Variable** | **Df** | **SS** | **MS** | ***F*** | ***P*** |
| --- | --- | --- | --- | --- | --- |
| **Initial inhibitory level** | 1 | 1.88e-8 | 1.88e-8 | 37.735 | <0.001 |
| **Initial inhibitory level:Interaction type** | 1 | 1.07e-9 | 1.06e-9 | 2.138 | 0.145 |
| **Initial inhibitory level:Combination** | 2 | 5.8e-10 | 2.92e-10 | 0.586 | 0.557 |
| **Residuals** | 217 | 1.08e-7 | 4.98e-10 |  |  |

We used a generalized linear model (GLM) to evaluate the effect of the initial inhibitory level of four selected drug combinations on adaptation rates, and additionally included interaction type and combination as nested factors.

Df: Degrees of freedom

SS: Sum of squares

MS: Mean sum of squares

*F*: *F*-ratio

*P*: *P*-value
