# Supplementary material for: Antibiotic combination efficacy (ACE) networks for a Pseudomonas aeruginosa model
Source: PLoS Biol. 2018 Apr 30;16(4):e2004356. doi: 10.1371/journal.pbio.2004356 (PMC5945231; doi:10.1371/journal.pbio.2004356)
Supplement: S4 Table — (DOCX) [file pbio.2004356.s015.docx]

**S5 Table. Effect test of the initial inhibitory level, interaction type and combination on the number of extinctions.**

| **Variable** | **Df** | **SS** | **MS** | ***F*** | ***P*** |
| --- | --- | --- | --- | --- | --- |
| **Initial inhibitory level** | 11 | 8.427 | 0.776 | 21.45 | <0.001 |
| **Initial inhibitory level:Interaction type** | 12 | 9.063 | 0.755 | 21.15 | <0.001 |
| **Initial inhibitorytreatment:Combination** | 24 | 1.5 | 0.063 | 1.75 | 0.017 |
| **Residuals** | 336 | 12 | 0.036 |  |  |

We used a logistic regression to evaluate the effect of the starting inhibition level of four selected drug combinations on extinction, including interaction type and combination as nested factors.

Df: Degrees of freedom

SS: Sum of squares

MS: Mean sum of squares

*F*: *F*-ratio

*P*: *P*-value
