# Supplementary material for: Antibiotic combination efficacy (ACE) networks for a Pseudomonas aeruginosa model
Source: PLoS Biol. 2018 Apr 30;16(4):e2004356. doi: 10.1371/journal.pbio.2004356 (PMC5945231; doi:10.1371/journal.pbio.2004356)
Supplement: S1 Data — (RTF) [file pbio.2004356.s016.rtf]

'Read me' first key to data files:

S2 Data: 

This data corresponds to the experiments evaluating drug interactions for 8 selected antibiotic pairs using the checkerboard approach and Bliss independence.

well = Position in the randomised 96-well plate.
time = Time at which optical density was taken. Measurements were taken every 15min for 12h.
combination = combination being evaluated
monotherapy1 = identity of one of the single-drugs
identifier_conc_mono_1 = categorical identifier for antibiotic 1 used for the design of the randomised plate. Each concentration is equivalent to a unique identifier. 
concentration_mono_1 = Concentration in ug/ml of the antibiotic identified as the single-drug 1.
monotherapy2 = identity of the second drug in the combination
identifier_conc_mono_2 = categorical identifier for antibiotic 2 used for the design of the randomised plate. Each concentration is equivalent to a unique identifier. 
concentration_mono_2 = Concentration in ug/ml of the antibiotic identified as the single-drug 2.
replicate = replicate per antibiotic concentration and combination, each was replicated 8 times.
od = Optical density (600nm).


S3 Data:

This data corresponds to the experiments evaluating drug interactions for 52 antibiotic combinations using the alpha determinant.

well = Position in the randomised 96-well plate.
time = Time at which optical density was taken. Measurements were taken every 15min for 12h.
od = Optical density (600nm).
drug_proportion = Drug proportion of the combined drugs. Values range from 0 (full dose of any drug A, set to IC75) to 1 (full dose of any drug B). Nine drug proportions were considered. Controls included a drug_proportion identified by 9 which is equivalent to a no-drug control and 10 to a no-drug and no-bacteria control. 
replicate = Replicate for each drug_proportion an combination. 9 replicates were included per drug_proportion and combination. 

S4 Data:

This data corresponds to the obtained optical density values during experimental evolution of 38 antibiotic concentrations. From this data we inferred growth rates and adaptation rates as specified in the Materials and Methods.

season = Season of evolution. There where 10 seasons of evolution, all in the presence of the antibiotics.
time = Time at which optical density was taken. Measurements were taken every 15min for 12h.
combination = Antibiotic combination used during experimental evolution.
mono1 = Antibiotic corresponding to the single-drug treatment 1.
mono2 = Antibiotic corresponding to the single-drug treatment 2.
drug_proportion = Drug proportion of the combined drugs. Values range from 0 (full dose of any drug A, set to IC75) to 1 (full dose of any drug B). Five drug proportions were considered. The no -drug control can be identified by a drug_proportion equal to 6.
replicate = Replicate for each drug_proportion an combination. Eight replicates were included per drug_proportion and combination. 
extinct = Boolean value determining whether the population was extinct (1) or not (0). 
od = Optical density (600nm).

S5 Data:

This data corresponds to the evolution experiment which assessed the influence of initial inhibitory levels 4 selected combinations on extinction frequencies and adaptation rates.

well = Position in the randomised 96-well plate.
season = Season of evolution. There where 10 seasons of evolution, all in the presence of the antibiotics.
time = Time at which optical density was taken. Measurements were taken every 15min for 12h.
combination = Antibiotic combination used during experimental evolution.
mono1 = Antibiotic corresponding to the single-drug treatment 1.
mono2 = Antibiotic corresponding to the single-drug treatment 2.
treatment = 0 corresponds to the no-drug control, 1 and 2 are the corresponding monotherapies, treatments 3-10 include different levels of inhibition starting from IC50 to >IC90, and 11 is the no-drug and no-bacteria control. 
extinct = Boolean value determining whether the population was extinct (1) or not (0). 
od = Optical density (600nm).
